# A Sensorless Modular Multiobjective Control Algorithm for Left Ventricular Assist Devices: A Clinical Pilot Study

**DOI:** 10.3389/fcvm.2022.888269

**Published:** 2022-04-25

**Authors:** Martin Maw, Thomas Schlöglhofer, Christiane Marko, Philipp Aigner, Christoph Gross, Gregor Widhalm, Anne-Kristin Schaefer, Michael Schima, Franziska Wittmann, Dominik Wiedemann, Francesco Moscato, D'Anne Kudlik, Robert Stadler, Daniel Zimpfer, Heinrich Schima

**Affiliations:** ^1^Center for Medical Physics and Biomedical Engineering, Medical University of Vienna, Vienna, Austria; ^2^Department of Cardiac Surgery, Medical University of Vienna, Vienna, Austria; ^3^Ludwig-Boltzmann-Institute for Cardiovascular Research, Vienna, Austria; ^4^Medtronic plc, Minneapolis, MN, United States

**Keywords:** left ventricular assist device (LVAD), mechanical circulatory support, physiological control, smart pumping, Valsalva maneuver, orthostatic transitions, submaximal bicycle ergometry

## Abstract

**Background:**

Contemporary Left Ventricular Assist Devices (LVADs) mainly operate at a constant speed, only insufficiently adapting to changes in patient demand. Automatic physiological speed control promises tighter integration of the LVAD into patient physiology, increasing the level of support during activity and decreasing support when it is excessive.

**Methods:**

A sensorless modular control algorithm was developed for a centrifugal LVAD (HVAD, Medtronic plc, MN, USA). It consists of a heart rate-, a pulsatility-, a suction reaction—and a supervisor module. These modules were embedded into a safe testing environment and investigated in a single-center, blinded, crossover, clinical pilot trial (clinicaltrials.gov, NCT04786236). Patients completed a protocol consisting of orthostatic changes, Valsalva maneuver and submaximal bicycle ergometry in constant speed and physiological control mode in randomized sequence. Endpoints for the study were reduction of suction burden, adequate pump speed and flowrate adaptations of the control algorithm for each protocol item and no necessity for intervention via the hardware safety systems.

**Results:**

A total of six patients (median age 53.5, 100% male) completed 13 tests in the intermediate care unit or in an outpatient setting, without necessity for intervention during control mode operation. Physiological control reduced speed and flowrate during patient rest, in sitting by a median of −75 [Interquartile Range (IQR): −137, 65] rpm and in supine position by −130 [−150, 30] rpm, thereby reducing suction burden in scenarios prone to overpumping in most tests [0 [−10, 2] Suction events/minute] in orthostatic upwards transitions and by −2 [−6, 0] Suction events/min in Valsalva maneuver. During submaximal ergometry speed was increased by 86 [31, 193] rpm compared to constant speed for a median flow increase of 0.2 [0.1, 0.8] L/min. In 3 tests speed could not be increased above constant set speed due to recurring suction and in 3 tests speed could be increased by up to 500 rpm with a pump flowrate increase of up to 0.9 L/min.

**Conclusion:**

In this pilot study, safety, short-term efficacy, and physiological responsiveness of a sensorless automated speed control system for a centrifugal LVAD was established. Long term studies are needed to show improved clinical outcomes.

**Clinical Trial Registration:**

ClinicalTrials.gov, identifier: NCT04786236.

## Introduction

Implantation of a Left Ventricular Assist Device (LVAD) is an established therapy for end-stage heart failure. Technological and patient management advances have resulted in continuously improving survival-rates. However, success of LVAD therapy is still limited by hemocompatibility associated adverse events and quality of life challenges (1).

Adaptation of the LVAD to the physiological demand of the patient, the final frontier of “smart pumping” has long been proposed as a potential contender in the race for better outcomes and quality of life. But this adaptation has arguably even regressed from the days of pulsatile fill-to-empty pumps (2).

Contemporary LVADs typically operate at a constant speed (CS), set by the VAD clinicians with only periodic check-ups (3). In CS, only the pump-specific pressure flowrate characteristic determines the function that maps pump flowrate to the difference between left ventricular and aortic pressure. The inherent conflation of afterload and preload is however quite different from the native control mechanism of cardiac output, which is much more preload sensitive, and much less afterload sensitive (4).

Short term fluctuations are not properly compensated by the pump-characteristic. Residual native adaptive mechanisms such as the Frank-Starling mechanism are often impaired but not completely absent in LVAD patients. Still, it has been shown that patients could benefit from additional pump support during activity (5, 6). On the other hand, patients were shown to exhibit high levels of ventricular suction, collapse of a ventricular structure onto the inflow cannula, often brought upon by unphysiologically low ventricular pressures, even when their CS set-speed has been optimally adjusted (7).

Thus, greater adaptation of pump support is warranted. This greater flexibility of pump support should ideally come within seconds, as quick hemodynamic changes are quite common in everyday life, such as while standing up or coughing. Other adaptations need to be made within minutes, or hours such as adaptation to prolonged activity or diurnal variations. Automatic physiologic control (PhC) promises to add this functionality to continuous flow LVAD.

Clinical application of PhC algorithms is still limited. While the HeartMate 3 (Abbott Laboratories, Chicago, IL, USA) reacts to Pulsatility Index events with transient speed decreases, all other deviations from constant speed in currently clinically used continuous flow devices such as the Lavare™ cycle (8) and the Artificial Pulse (9), are periodically triggered and thus not adaptive to patient demand.

Previous research efforts have identified numerous control strategies which have been validated *in silico, in vitro, ex vivo*, but only once in a clinical trial (10, 11). In these studies, it could be shown, that these controllers adapt rapidly to changes in patient state, such as changes in venous return, arterial resistance, and simulated exercise (12, 13).

In a previously conducted clinical trial, it could be shown that a PhC algorithm could safely increase flowrate while decreasing pulmonary capillary wedge pressure during ergometry. The used pump system was an axial pump system with included flowsensor (14).

In this paper, an adapted, modular sensor-less multi-objective controller for a centrifugal pump (Medtronic HVAD) is described and tested in a clinical pilot study.

## Methods

### Data Availability

Additional data can be found in the Supplementary Material. Data in accordance with privacy and confidentiality restrictions is available from the corresponding author upon request.

### Patients

After approval was obtained from the institutional review board of the Medical University of Vienna and from the Austrian National Authority for Medical Devices (BASG), six patients who received an HVAD at the Medical University of Vienna were enrolled and completed their measurements between December 2020 and June 2021, when the study was terminated, due to the global stop of sale of the Medtronic HVAD. The study was registered (Clinicaltrials.gov: NCT04786236) and informed consent was provided by the patients. Pre-test screenings included echocardiographic evaluation to assess functional status of the ventricles and to rule out intraventricular or aortic root thrombus. Home documentation and lab results were checked for proper anticoagulation. Patients with history of stroke or suspected pump thrombosis as well as patients with known coagulopathies were excluded.

### Study Design

The single blinded, crossover study protocol consisted of a set of activities performed twice, once in CS mode, and once in PhC mode. The sequence of the speed modes was randomized via permuted block randomization. Measurements were performed either at the intermediate care (IC) unit, or during outpatient follow up in the ambulatory ward (AM).

#### Activity Protocol

Patients performed activities adapted to their capabilities. The standardized protocol consisted of three segments.

#### Orthostatic Changes

Patients performed postural transitions from a supine position to standing position and in the reverse direction. If this was not possible for them, a transition from supine to the sitting position was performed. For analysis, the transitions were subdivided into 3 phases: Steady state (SS = 5–10 s before transition initiation), initial phase (IP = 0–15 s after transition initiation) and late phase (LP = 15 – 60 s after initiation) (15).

#### Valsalva Maneuver

While seated, patients forcefully exhaled into a positive expiratory pressure device (BA-Tube, Flores Medical GmBH, Germany) for up to 15 s. For analysis, the maneuvers were again subdivided into a steady state (SS), straining phase (SP), and recovery phase (RP). SS was again defined as 5 s before initiation of strain. SP comprises phase I and phase II from conventional Valsalva classification: the period of forced exhalation. RP is defined as phases III and IV, or recovery back to baseline. This was defined as the first 30 s after strain release (16).

#### Submaximal Ergometry

Patients completed a ramped submaximal ergometry (ER) protocol (Daum Electronics Gmbh, Fürth, Germany). If possible, initial load was set to 20 Watts and increased each minute until 70% of the maximum power during ergometry in their latest maximal spiro-ergometry was achieved. If 20 Watts exceeded the capabilities of the patient a bed pedaling device was used instead. An additional 1-minute warm-up and cool down period was optional. For analysis, ER is subdivided into a warmup phase (WU: First 10% of total duration), an early phase (EP: 10–60%), a late phase (LP: 60–100%) and a cooldown phase (CD) of 1 min.

OR and VA were repeated three times for each control mode and outcome measures were averaged.

### Control System

**Figure 1:**
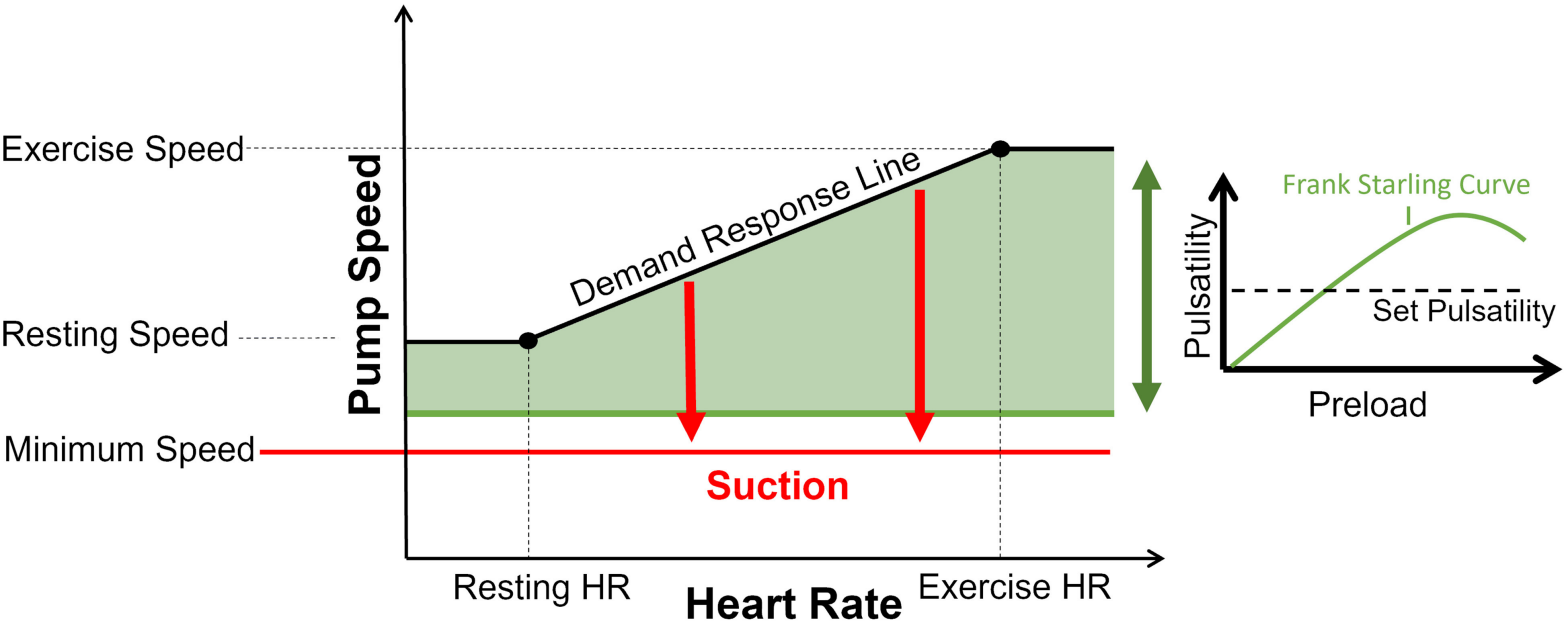
Graphic representation of the control algorithm. The demand response submodule sets an upper speed limit based on heart rate. The flow pulsatility is partially governed by the Frank Starling curve of the ventricle (in the right panel). If flow pulsatility exceeds the set-point, sufficient filling is expected and speed is increased proportionally up to the demand response line (black). Conversely, if flow pulsatility is below set-value, speed is decreased, eventually down to the minimum speed limit. If suction is detected, speed is reduced by discrete steps until suction is cleared. If speed is decreased to minimum speed (red line) and suction is still present, speed is not further decreased. Speed is increased once suction is no longer present.

The developed PhC system consists of 3 hemodynamically functional modules and one supervision module. An overview is given in Figure 1. The demand response module (DR) defines a linear function of heartrate (HR) to desired maximal speed. HR is estimated from the pump current and gradual changes are considered for the module whereas sudden changes are seen as pathologic and ignored. A pulsatility presuction module (PS), aims at maintaining a constant flowrate pulsatility, if not limited by other modules. A suction reaction module (SU) detects suction and reacts by incrementally reducing speed. Finally, a supervision module arbitrates conflicting commands, and a rate limiter (RLI) module enforces relative and absolute speed limits. A comprehensive description can be found in Supplementary Material S1.

### Hardware and Software Setup

**Figure 2:**
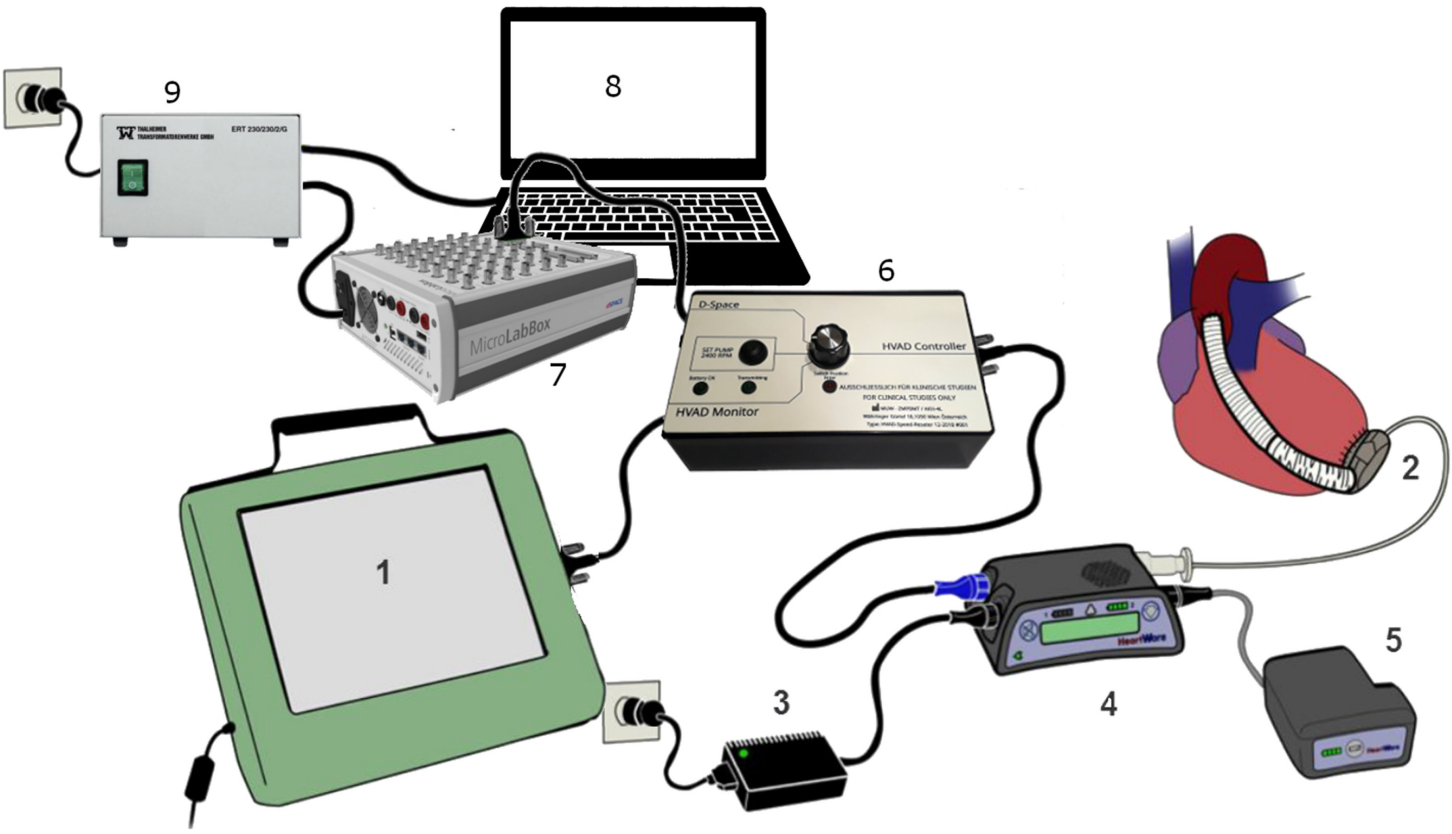
The clinical routine hardware setup of the HVAD (1–5) was modified with the addition of a switchbox (6), the dSpace MicrolabBox (7), a laptop (8), and an isolation transformer (9). The switchbox routes serial transmission to the controller between the monitor, the internal microprocessor and the dSpace based system (1: Monitor; 2: HVAD pump; 3: Power supply; 4: Controller; 5: Battery; 6: Switchbox; 7: dSpace Microlabbox; 8: Laptop; 9: Isolation Transformer). Adapted from (8).

PhC algorithms were developed for a centrifugal LVAD (HVAD, Medtronic, Figure 2, 1-5). These algorithms were implemented in Simulink/Matlab (MathWorks INC, Natick, MA, USA) and compiled onto a prototyping unit (Figure 2, #7) (MicroLabBox, dSPACE GmbH, Paderborn, Germany) which exchanges information with the laptop (Figure 2, #8). The laptop displays a custom graphical user interface. The processing unit sends and receives data from the controller (Figure 2, #4) via the serial data port. A switchbox is implemented as a safety measure (Figure 2, #6). It allows quick switching of the source of speed commands between standard manual operation via the clinical monitor (Figure 2, #1) and the processing unit.

### Recorded Data

Pump power, current, speed and estimated flowrate were recorded at 50 Hz, which were then also used to calculate derived indices such as Aortic Valve opening (17), Suction Detection (18), and HR (19). For greater arrhythmia-detection accuracy, patients were additionally outfitted with a 5-lead Holter electrocardiographic (ECG) device (medilog® AR 12 plus, Schiller AG, Baar, Switzerland). RR Intervals were detected by the Pan-Tompkins algorithm (20) and arrhythmias were classified via the Medilog Adapt Algorithm (21). Finally, patient demographic data were extracted from the hospital database and inflow cannula angles were measured as described in (22).

### Setpoint Design

CS speed was not modified from the pre-test setpoint established by the routine clinical team, which aims to maintain a neutral septum position and, if possible, intermittend aortic valve opening. Speeds are confined to the recommended range of 2,400–3,200 rpm in CS operation.

For PhC, HR range was determined by reviewing retrospective logfile recordings when available and selecting the 10th and 90th HR percentile. Otherwise, baseline HR was set to resting HR during pretest sitting and 30 bpm added for exercise HR. Speed range for DR was set to 200 rpm below and 200 above pre-test CS set speed for rest and exercise set speed, respectively.

Set pulsatility was set to 2 L/min below usual pulsatility, determined by logfile recordings and during premeasurement evaluation but at a value not smaller than 1 L/min.

The setpoints were set by the attending investigator, the values above served as guidelines, but could be overruled, thus in some patients, DR setpoints or the PS Setpoint were set deliberately high to achieve more PS contribution.

### Statistical Analysis

All values are stated as: median and [interquartile range] or mean ± standard deviation.

## Results

### Demographics

A total of six patients partook in the investigation for a total of 13 measurements. A demographic overview can be found in Supplementary Table S2-1. All patients were male. All but one patient were on beta-adrenergic blocking agents and all but two received ACE Inhibitors, while three were on amiodarone. Patient 5 had grade II aortic insufficiency at baseline echocardiographic evaluation. Coronary cannula inflow angles ranged from −30° to 32°.

### Overview of Tests

Three patients completed 3 tests, one patient 2 tests and two patients completed only one session for a total of 13 tests. Three tests took place at the IC and the remaining 10 at AM. In 10 measurements, the patients were able to complete the entire protocol. In 2 tests, patients were not able to perform ER and in 2 tests at the IC, ER was performed with the bed pedal exerciser. In 2 tests comparable data for VA could not be collected. For OR, in 8 tests patients could safely stand up, in 4 patients could sit up. In 1 test no comparable OR could be collected.

Pre-test sitting echocardiography confirmed partial assistance in 8/13 tests. CS pump settings averaged 2682 ± 79 rpm. Baseline HR was 76 ± 16 bpm and mean arterial pressure was 79 ± 10 mmHg. Pump flow was 4.9 ± 0.4 L/min and pulsatility was 3.5 ± 0.6 L/min. Supplementary Table S2-2 provides a detailed overview of the tests.

### Setpoints

Six out of 13 tests were performed with setpoints as described in setpoint design. In 7/13, SU and PS modules were focused due to either increased DR setpoints (3/7) or pulsatility setpoints at or above baseline pulsatility (4/7) (see Supplementary Table S2-2).

### Safety Outcomes

Throughout the entire study, there was no need for any safety switchover intervention. Neither CS nor PhC mode posed any risk to the patient as judged by the attending clinician. There were no study related adverse events.

### Aggregate Comparative Results for Standardized Protocol

**Figure 3:**
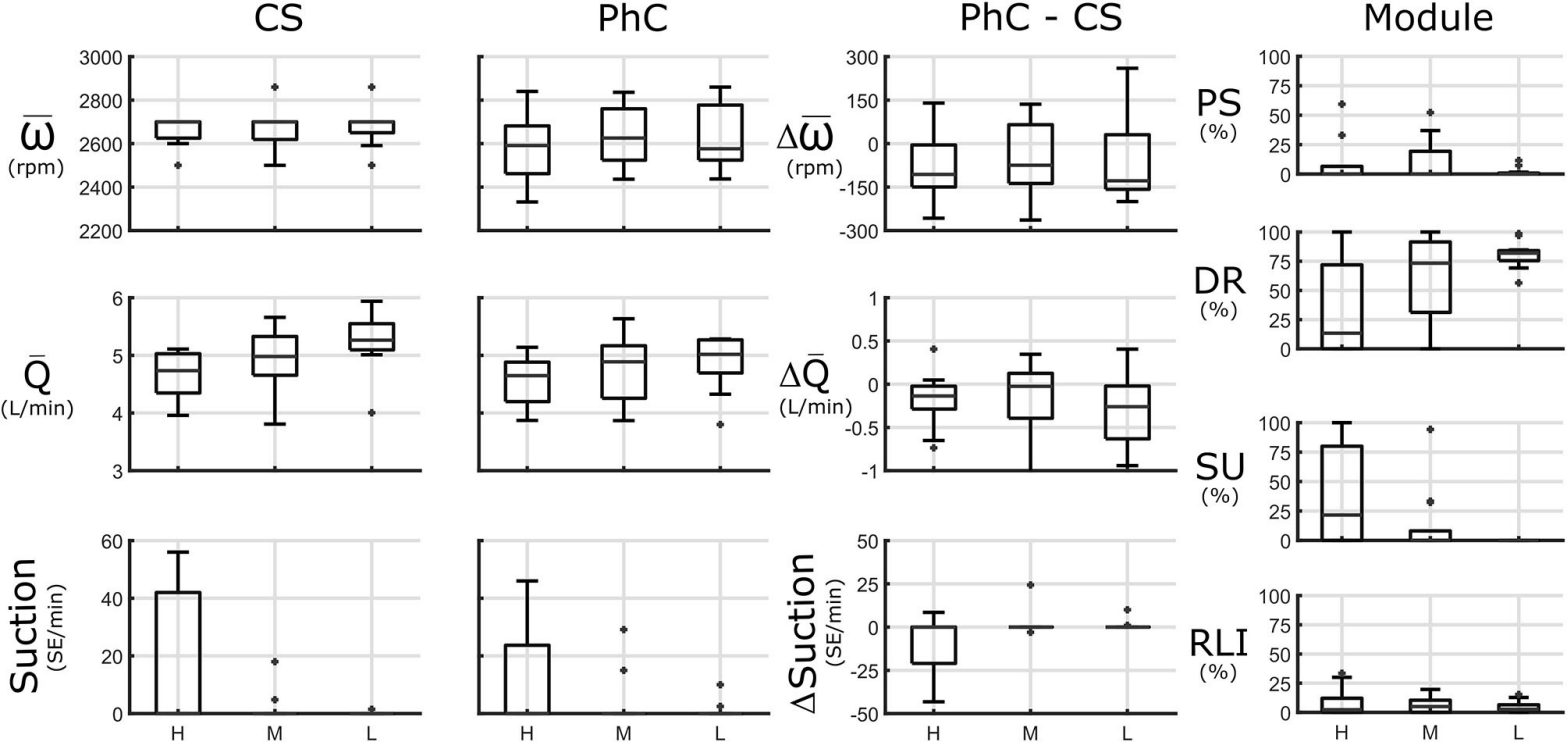
Steady state posture differences between standing(H), sitting (M), supine (L). Left panels: Speed (ω), flowrate (Q), and suction [in Suction Events (SE)/minute] in constant speed (CS) and physiologic control (PhC) and their differences. Module panel: control module activation for pulsatility (PS), demand response (DR), suction response (SU), and rate limited increase (RLI) modules. Speed is reduced in all postures in PhC modes compared to CS resulting in reduced flowrates and reduced suction burden during standing. DR module mainly governs set speed in supine and sitting position with increasing contributions from the SU modules while standing.

**Figure 4:**
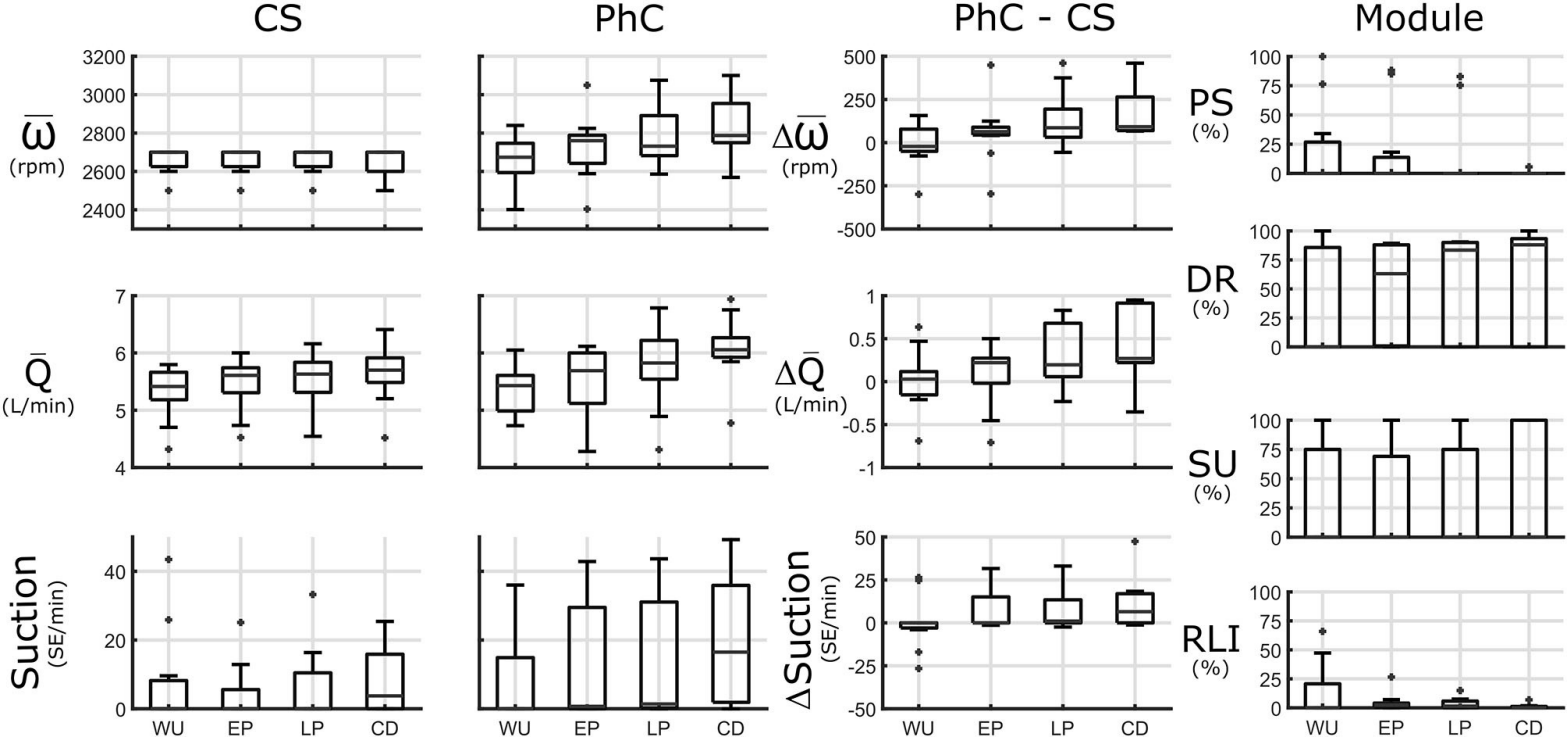
Orthostatic transitions from the supine position to either sitting or standing. Left panels: Speed (ω), flowrate (Q), and suction [in Suction Events (SE)/minute] in constant speed (CS) and physiologic control (PhC) and their differences are presented in steady state (SS) initial transition phase (IP) and late phase (LP). Module panel: control module activation: activation for pulsatility (PS), demand response (DR), suction response (SU), and rate limited increase (RLI) modules. Speed is reduced in PhC compared to CS in all phases. Lower suction burden in IP and LP is observed. There is predominant DR activation at SS with increasing contributions of the PS and SU module at later stages of the transition.

**Figure 5:**
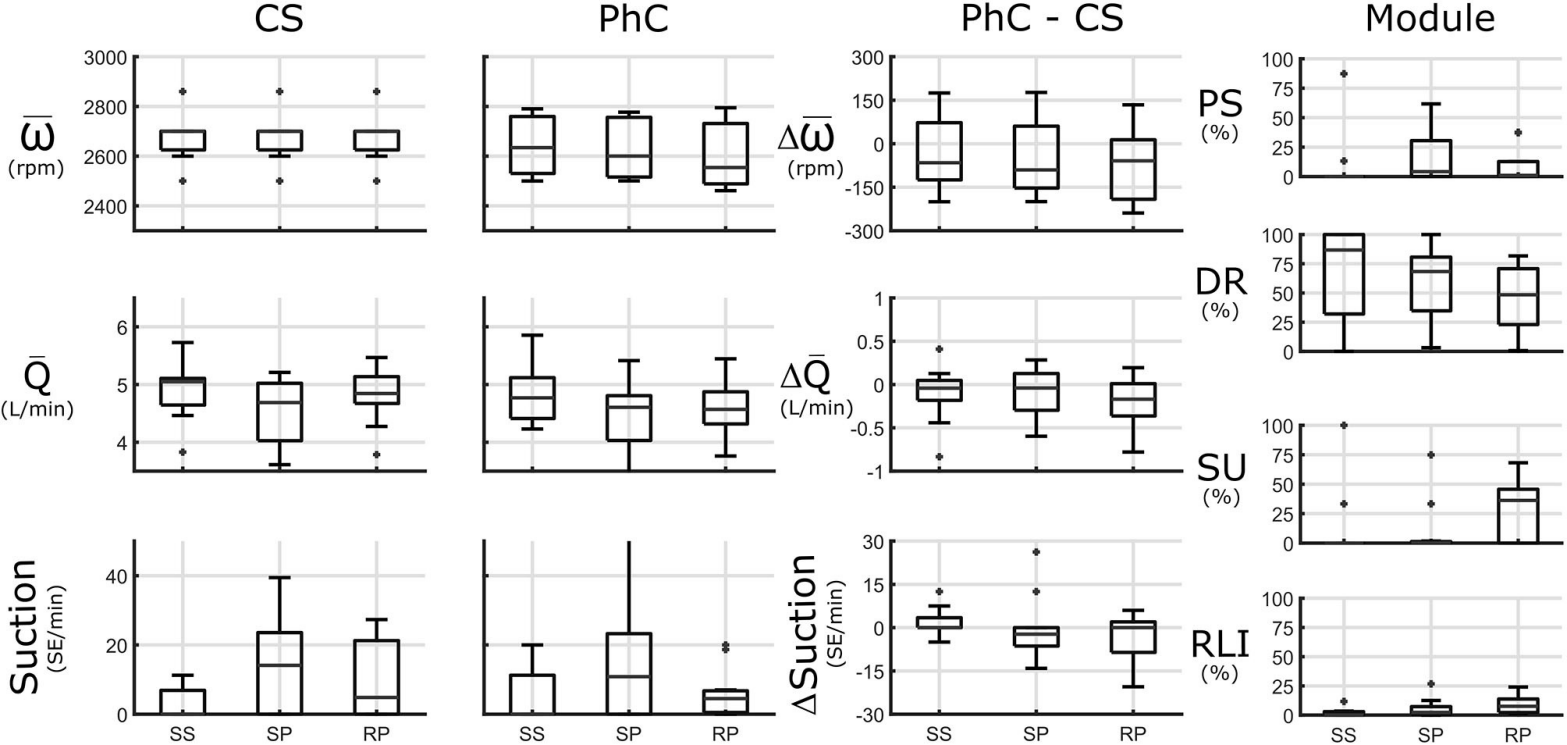
Valsalva maneuver (VA) is subdivided into 3 phases: steady state (SS), strain phase (SP) and recovery phase (RP). Left panels: Speed (ω), flowrate (Q), and suction [in Suction Events (SE)/minute] in constant speed (CS) and physiologic control (PhC) and their differences are presented. Module panel: control module activation: activation for pulsatility (PS), demand response (DR), suction response (SU), and rate limited increase (RLI) modules. Speed is reduced in PhC compared to CS, resulting in lower suction burden.

**Figure 6:**
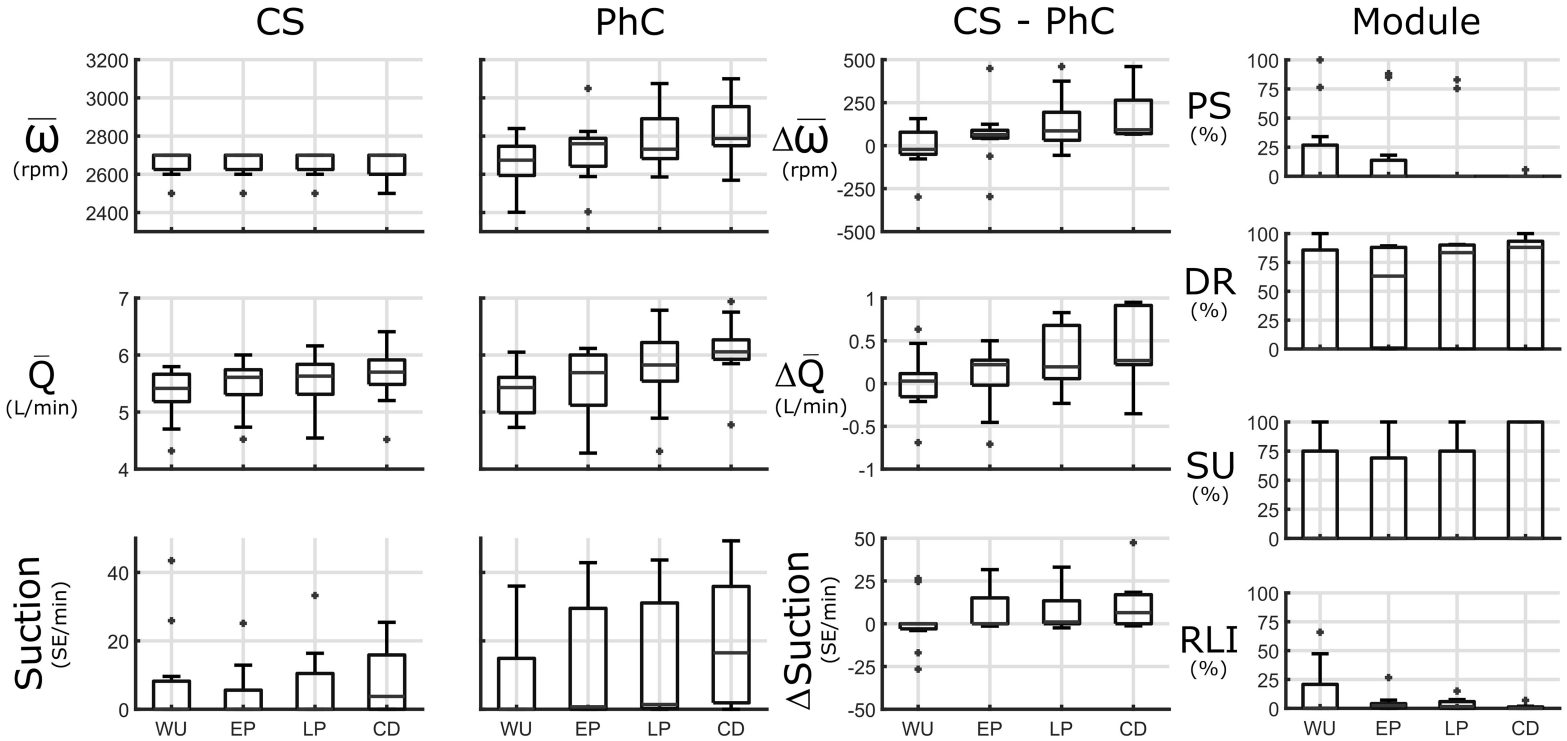
Submaximal ergometry is subdivided into 4 phases: warm up (WU), early phase (EP), late phase (LP) and cool down (CD). Left panels: Speed (ω), flowrate (Q), and suction [in Suction Events (SE)/minute] in constant speed (CS) and physiologic control (PhC) and their differences are presented. Module panel: control module activation: activation for pulsatility (PS), demand response (DR), suction response (SU), and rate limited increase (RLI) modules. Speed during ergometry is increased in PhC compared to CS resulting in increased pump flowrates, especially at later stages of submaximal exercise.

This section reports on the average differences between the PhC and CS. Figures 3–6 provide an overview over the single standardized interventions. A per-test summary as well as a comprehensive collection of all the single snapshots can be found in the Supplementary Materials S3, S4-6, respectively.

### Orthostatic Changes (OR)

#### Orthostatic Steady State

In CS pump-flowrate was highest in supine position at 5.3 (5.1, 5.5) L/min with reduced flowrate at sitting position at 5.0 (4.7, 5.3) L/min and lowest flowrate in standing posture [4.6 (4.2, 5.0) L/min]. Suction occurred in 5/10 sessions in standing, and in 2/13 in sitting.

PhC-mode resulted in a lower pump speed compared to CS and was lowest in supine [−130 (−150, 30) rpm] and similar for sitting [−75 (−137, 65) rpm] and standing [−79 (−150, −4) rpm]. Resulting in minor flowrate reductions in standing and supine posture but not during sitting.

Suction burden in standing posture was reduced in PhC compared to CS in 4 of the 5 tests, where suction occurred [0 SE/min (−18, 0)]. However, during most measurements patients did not experience suction in either mode.

Speed was mainly governed by DR in supine [81% (71, 84)] and sitting [73% (31, 92%)]. While standing, SU module was increasingly activated. See Figure 3 for an overview and Supplementary Figure S3-1 for an extended overview.

#### Orthostatic Transitions

In CS, flowrate increased from the baseline of 5.2 (5.0, 5.5) L/min at steady state to 5.5 (5.0, 5.7) in the initial phase due to increase in diastolic flow. In LP flowrate was decreased below baseline to 5.0 (4.4, 5.4) L/min. Suction occurred either in the initial phase (5/12) or in the late phase (6/12).

In PhC, speed was already reduced by −136 (−169, 30) rpm during steady state. From there, speed was slightly increased in the initial phase to −112 (−155, 51) rpm and further increased in late phase to −42 (−152, 47) rpm. This led to reduced flowrates in all phases compared to CS.

Suction in initial phase was decreased in 3/5 sessions and increased in 1/5 compared to CS. In the late phase it was reduced in 3/5 sessions and increased in 2/5. In the two tests with increased suction prevalence in late phase in PhC, speed was either set higher than speed set speed already at steady state (test 12) or repeated attempts at speed increase at low speeds (<2,500 rpm) retriggered suction events (test 9).

From predominant DR activation in steady state, increased activation of SU and PS (Contribution >20% in 4/12 tests) as well as the RLI module is recorded in the initial phase and late phase. In late phase, DR governed speed even less. High activation of PS in test 6 prevents suction without activation of the SU module. SU and RLI modules were also increasingly activated in the late phase. See Figure 4 for an overview and Supplementary Figure S3-2 for an extended overview. The mentioned single snapshots can be found in Supplementary Material S4.

### Valsalva Maneuver

In the straining phase in CS, flowrate was reduced from a steady state of 5.0 (4.6, 5.1) L/min to 4.7 (4.0, 5.0) L/min due to suction events, which occurred in 7/11 sessions for a median suction burden of 14 (0, 24) SE/min, and persisted at least for some additional time after release in the recovery phase for a median suction burden of 5 (0, 21) SE/min. In 4/11 sessions there was almost no flowrate response during VA (example: test 3 in Supplementary Material S-5).

In PhC, upon straining, the decreased speed of steady state is mostly upheld or slightly reduced compared to CS [−90 (−153, 61)] rpm resulting in similar flowrates to CS. This led to reduced suction burden in the straining phase compared to CS for 5/7 Tests for a median reduction of −2 (−6, 0) SE/min (example: test 10), in the 2 tests with increased suction burden (test 9 and 13 in Supplementary Material S-5), suction occurred at low speeds (<2,600 rpm) or speed was already greatly increased at steady state due to pulsatility setpoint strategy and insufficient speed decrease before suction. Suction burden in recovery phase was reduced in PhC compared to CS in 5/11 Tests [0 (−9/2), SE/min].

DR-Module was again predominant during steady state. In straining phase PS gained relevance in 5/11 tests. Finally in recovery phase, SU was activated in 6/11 tests. See Figure 5 for an overview and Supplementary Figure S3-3 for an extended overview. The mentioned single Valsalva maneuver snapshots can be found in Supplementary Material S5.

### Ergometry

Due to the activity involved in getting on the ergometer and starting movement, warmup flowrate in CS was already increased compared to sitting at rest by 0.4 L/min to 5.4 (5.2, 5.7) L/min. Only in 5/11 tests, flowrate further increased throughout the duration of ergometry by more than 0.3 L/min. The flowrate increase from warmup to late phase was 0.2 (0.0, 0.4) L/min. In 5/11 tests patients experienced suction events, especially in late phase and cooldown.

In PhC, speed at warmup was within 100 rpm of CS settings in 8/11 tests for a medium difference of −21 (−50, 78) rpm. It was increased by at least 100 rpm in early phase or late phase for 8/11 tests for a median increase of 64 (44, 89) and 86 (31, 193) rpm, respectively. In patients, where upper limits of the DR Module were set higher, speed was increased by up to 500 rpm compared to CS (tests 11 and 13). Persistent suction upon speed increase (test 9) or a complete chronotropic incompetence and lack of pulsatility response shortly after implantation (test 1 and 4) restricted speed increase. In one test, speed was only increased in the late phase, as pulsatility remained low until then (test 8).

From warmup to late phase, flowrate was increased by more than 0.5 L/min in 5/11 tests for an overall median increase of 0.2 (0.1, 0.8) L/min. In 3/11 tests flowrate in late phase was increased by ~0.9 L/min compared to CS.

Suction burden was increased in PhC compared to CS in 4/11 tests in early phase by 0 (0, 15) SE/min and late phase by 1 (0, 13) and 5/11 in the cooldown phase by 6 (0, 17) SE/min. There was no marked decrease in flowrate pulsatility before suction in these patients (minimum pulsatility > 3.5). The reasons for the suction increase were suction classifier discrepancy (test 2), or repeated unsuccessful attempts at speed increase past CS setpoint in test 9 and 12, which were by pat 4 and 2 with cannula inflow angles of 32° and −15°, respectively. In the 2 tests where speed was increased the most (>300 rpm) (tests 11 and 13) suction burden remained below 3 SE/min in both modes. In these patients (Pat. 1 and 5) cannula angles were more favorable at 20° and 9°, respectively.

During warm up the speed increase was rate limited by the RLI at least partially in 6/11 tests. In 3/11 tests pulsatility was not always sufficient to increase speed and in another 3/11 tests repeatedly encountered suction events triggered activity of the SU module. Speed was increasingly set based on the DR module with longer duration of exercise. See Figure 6 for an overview and Supplementary Figure S3-4 for an extended overview. the mentioned submaximal exercise snapshots can be found in Supplementary Material S6.

### Rhythmological Summary

In 4/7 tests arrhythmias such as non-sustained ventricular tachycardia were recorded during OR (3 tests), VA (4 tests), and ER (2 tests). All 38 tachycardia episodes (30 of which occurred in test 12), were triggered by an immediately preceding suction event. However, not every suction event triggered arrhythmic episodes.

Arrhythmia duration was increased in PhC mode in ER in 2 Sessions (CS: 3.5 s, PhC: 13.2 s of arrhythmia). It was decreased in OR in 2 patients and increased in one. In VA unchanged in one test, reduced in two and increased in one session. In one session arrythmia only occurred in CS mode.

## Discussion

A multi-objective physiological control algorithm was developed and tested in a safe testing environment in a clinical pilot trial. The controller was able to modulate pump support without the use of additional sensors.

### Controller Performance Overview

The controller set lower pump speeds, compared to CS in inactive patient states, such as supine and sitting posture. This led to a lower baseline speed during VA and OR, contributing to reduced suction burden in these suction-prone maneuvers. However, if speed was higher than baseline, speed decreases were generally too slow to avoid suction.

During ER speed was increased by up to 500 rpm compared to CS, generating higher pump flow. However, in other sessions, speed could not be increased above CS speed due to recurrent suction, even with arguably sufficient venous return.

### Suction Reduction

Lower pump speed correlated with lower suction events, and suction burden could be reduced in some patients. In the presented dataset every single episode of arrhythmia was preceded by a suction event, similarly to previous experience (23). However, the exact impact of arrhythmia on patient outcomes is not fully understood. While transient episodes of arrhythmia seem to be well tolerated without greater risk for syncope or sudden cardiac death, a non-contractile or fibrillating RV might increase risk for thrombosis (24). Higher pump speeds have previously also been correlated with reduced aortic valve health (25), increased thrombogenicity (26), and increased bleeding (27), partially by increasing pulsatility. However, no definitive study so far has directly clinically linked suction to worse outcomes, possibly due to its low visibility (7).

### Increased Support During Activity

On the other side of the spectrum, speed increase during activity has previously been shown to reduce PCWP (28) and improve submaximal capacity (6). It was shown that insufficient speed during exercise reduced peak cardiac output and peak oxygen consumption (29). In our collective we have shown that speed increases during ergometry can automatically be achieved for most patients. In some patients however, recurrent suction or lack of pulsatility does not permit speed increases.

### Evaluation of Control Modules

The heart-rate driven DR module was the most activate module. Only in 3 tests HR range was <10 bpm. Two of these tests were within the first 20 post-operative days and one in a patient with multiple comorbidities that relied on a wheelchair. HR tended to be quite responsive during OR and early phases of ER.

The PS module was designed to become active at low ventricular preloads. Only in patient one pulsatility of <0.5 L/min could be achieved upon full unloading. In all other patients there remained considerable residual pulsatility before suction, sometimes without any decrease prior to onset, or a very rapid decrease, limiting the utility of the PS module. However, especially at the IC it was observed that pulsatility was quite responsive to posture changes, such that supine posture produced the highest pulsatility and standing posture the lowest, possibly due to venous pooling in the lower extremity. A pulsatility based controller will thus increase speed upon lying down, which is in line with preload-based paradigms.

In 2 of the 6 patients (33%) speed could not markedly be increased from the CS set speed even at later stages of ergometry, with arguably sufficient venous return. Here, geometrical challenges such as the cannula position, a small LV cavity or restricted filling might be prohibitive to speed increases by restricting pulsatility or triggering suction. Rapid onset of suction in the selected maneuvers often did not leave sufficient time for reaction when speed was already increased, however in some cases, speed reductions were able to break the “vicious cycle” of suction (7).

Absolute and relative speed restrictions were implemented for safety reasons but limited the control system, resulting in excessive speed if baseline speed was already higher at maneuver onset. RLI did not greatly delay PhC reaction to exercise.

### Limits of Physiological Control

Non-optimal inflow cannula position, as it may happen if the patient gains weight after implantation, or small cavity size, can trigger suction even when venous return is otherwise sufficient. In our collective we observed suction even at low speeds of 2300 rpm.

Great interpatient variability needs consideration when adapting PhC algorithms. As shown here and also previously by Jain et al. (30) patient hemodynamic responses are highly variable during standardized maneuvers, for example due to baroreflex failures or different baseline preloads. Insufficient vasomotor tone resulting in excessive venous pooling could also explain some of the large responses especially at the IC to orthostatic transitions. Additionally, changes in medication such as omission of betablockers in patient 3 greatly changed hemodynamic response.

### Differences to Previous Studies

Differences to previous implementations of the control algorithm in the axial Micromed Debakey LVAD, are the centrifugal pressure-flow characteristic of the HVAD (14, 31). Thus, pulsatility is more influenced by hyper- and hypotension and speed changes as the position on the HQ curve becomes more important due to the non-linearity. The pump-characteristic also causes higher pulsatility in lower flowrates. Additionally, differences in implantation techniques to previous devices might cause a higher rate of positional suction.

### Implementation in Other Pump Systems

While the algorithms were only implemented in the HVAD in this study, feasibility of application to other pump systems ensures that progress is not lost with the discontinuation of this specific device. The control algorithms consist of modules that may be adapted for other systems. A suction detection module can be developed either by accessing motor-information of the pumps or with additional sensors within the ventricle or in the pump. Even without the high temporal resolution of 50 Hz provided by the HVAD, many suction detection features rely only on averages and extrema (18). Similarly, the pulsatility module requires only the signal extrema readily available in the HeartMate 3™ (Abbott Laboratories, Chicago, IL, USA) for example, allowing rapid translation. The cycle detection algorithm required for the DR module benefits from higher sample rates. However, all currently available devices are based on similar DC motor technology and thus have access to motor signals such as current and speed, however not all devices provide this information via a serial interface like the HVAD.

The sensorless control algorithms may even be used in systems with additional sensors, either as a redundant safety mechanism against sensor failure or to supplement additional information. Pressure sensor, placed either at the cannula tip, atrium or pulmonary artery could give important insight into patient hemodynamics, and especially about preload. However, current devices like the Cardio MEMS (Abbott Laboratories, Chicago, IL, USA) could not register all types of suction and could not detect ventricular contraction due to its placement in the pulmonary artery.

### Limitations

Due to the nature of the pilot study, rigid setpoint protocols were not upheld, and setpoint options were explored, resulting in additional data heterogeneity. Furthermore, low patient numbers due to the discontinuation of the HVAD device, without the inclusion of female subjects, a short observation period, lack of invasive hemodynamic monitoring as well as a protocol not geared toward investigating physiological quantitative differences restricts conclusions drawn from the study.

### Outlook

Due to the discontinuation of HVAD sales, follow-up studies with this implementation of the controller are not planned. Very similar algorithms have previously demonstrated efficacy in an entirely different pump system and due to the sensorless nature the presented control concepts are easily translated to additional other continuous flow pumps, such as the HeartMate 3™, Evaheart®2 (Sun Medical Technology Research Corp., Nagano, Japan) or even the family of Impella® pumps (Abiomed Inc., Danvers, Ma.), as well as other pump systems that rely on a DC motor.

## Conclusion

A clinical pilot trial showed feasibility of a system for physiological control for a contemporary left ventricular assist device. It showed that support could be increased during physical exertion and decreased upon scenarios of overpumping, thereby decreasing suction burden and the correlated arrhythmias. Further study is needed to investigate how this closer adaptation to physiologic demand translates to long term patient outcomes.

## Data Availability Statement

The original contributions presented in the study are included in the article/Supplementary Material, further inquiries can be directed to the corresponding author.

## Ethics Statement

The studies involving human participants were reviewed and approved by Ethikkommission Medizinische Universität Wien. The patients/participants provided their written informed consent to participate in this study.

## Author Contributions

The study was conceptualized, designed, and managed by HS and MM. Original manuscript draft was written by MM. Primary data analyses were done by MM and GW. The control algorithm was developed by MM, CG, D'AK, RS, and HS and preliminary tests were performed by MM, TS, and PA. TS and CM were responsible for patient screening and supervision. Investigations were performed by MM, TS, and HS with CM, A-KS, FW, DW, and DZ performing medical investigations. Regulatory responsibilities were shared by MM, DZ, and HS. Infrastructure was provided by HS and DZ. Additional supervision and manuscript editing was provided by FM. All authors reviewed and approved the final version of the submitted manuscript.

## Funding

This study received funding from the Ludwig Boltzmann Institute for Cardiovascular Research and an External Research Project grant by Medtronic plc. The funder: Medtronic had the following involvement with the study: Manuscript editing. The funder was not involved in the study design, collection, analysis, interpretation of data or the decision to submit it for publication.

## Conflict of Interest

This study received funding from the Ludwig Boltzmann Institute for Cardiovascular Research and an External Research Project grant by Medtronic plc. The funder: Medtronic had the following involvement with the study: Manuscript review. The funder was not involved in the study design, collection, analysis, interpretation of data or the decision to submit it for publication. TS, Disclosure: Consultant, Advisor (Abbott, Medtronic); Research Grant (Abbot, Medtronic). DW and DZ, Disclosure: Advisor, Proctor, Research Grants, non-financial Support, Speaker Fees (Abbott, Medtronic, Berlin Heart, Edwards). HS, Disclosure: Research Grant, Advisor (Medtronic). D'AK and RS were employed by Medtronic plc. The remaining authors declare that the research was conducted in the absence of any commercial or financial relationships that could be construed as a potential conflict of interest.

## Publisher's Note

All claims expressed in this article are solely those of the authors and do not necessarily represent those of their affiliated organizations, or those of the publisher, the editors and the reviewers. Any product that may be evaluated in this article, or claim that may be made by its manufacturer, is not guaranteed or endorsed by the publisher.

## Abbreviations

AM: Ambulatory ward
CS: Constant speed
DR: Demand response module
ER: Submaximal ergometry
HR: Heart Rate
IC: Intermediate care unit
IQR: Interquartile range
LVAD: Left Ventricular Assist Device
OR: Orthostatic changes
PhC: Physiological control
PS: Pulsatility-presuction module
RLI: Rate limited increase module
SU: Suction detection and reaction module
VA: Valsalva maneuver.

## References

[1] Teuteberg JJ Cleveland JC Cowger J Higgins RS Goldstein DJ Keebler M. The society of thoracic surgeons intermacs 2019 annual report: the changing landscape of devices and indications. Ann Thorac Surg. (2020) 109:649–60. 10.1016/j.athoracsur.2019.12.005 32115073

[2] Fukamachi K Shiose A Massiello A Horvath DJ Golding LAR Lee S. Preload sensitivity in cardiac assist devices. Ann Thorac Surg. (2013) 95:373–80. 10.1016/j.athoracsur.2012.07.077 23272869 PMC3592985

[3] Schlöglhofer T Robson D Bancroft J Sørensen G Kaufmann F Sweet L. International coordinator survey results on the outpatient management of patients with the heartware® ventricular assist system. Int J Artif Organs. (2016) 39:553–7. 10.5301/ijao.5000538 28058699

[4] Lim HS Howell N Ranasinghe A. The physiology of continuous-flow left ventricular assist devices. J Card Fail. (2017) 23:169–80. 10.1016/j.cardfail.2016.10.015 27989869

[5] Gross C Marko C Mikl J Altenberger J Schlöglhofer T Schima H. Pump flow does not adequately increase with exercise. Artif Organs. (2019) 43:222–8. 10.1111/aor.13349 30155903 PMC6589923

[6] Jung MH Houston B Russell SD Gustafsson F. Pump speed modulations and sub-maximal exercise tolerance in left ventricular assist device recipients: a double-blind, randomized trial. J Hear Lung Transplant. (2017) 36:36–41. 10.1016/j.healun.2016.06.020 27522486

[7] Gross C Schima H Schlöglhofer T Dimitrov K Maw M Riebandt J. Continuous LVAD monitoring reveals high suction rates in clinically stable outpatients. Artif Organs. (2020) 44:E251–62. 10.1111/aor.13638 31945201 PMC7318142

[8] HeartWare I. HeartWare Ventricular Assist System. (2012). p. 1–104. Available online at: https://www.heartware.com/sites/default/files/uploads/docs/ifu00001.21_us_pma_ifu_060815.pdf (accessed November 13, 2021).

[9] Corp. T. HeartMateIII Instructions for Use. (2017). Available online at: https://www.accessdata.fda.gov/cdrh_docs/pdf16/P160054C.pdf (accessed November 14, 2018).

[10] Tchantchaleishvili V Luc JGY Cohan CM Phan K Hübbert L Day SW. Clinical implications of physiologic flow adjustment in continuous-flow left ventricular assist devices. ASAIO J. (2017) 63:241–50. 10.1097/MAT.0000000000000477 28459742

[11] Maw M Moscato F Gross C Schlöglhofer T Schima H. Novel solutions for patient monitoring and mechanical circulatory support device control. In: Karimov JH, Fukamachi K, Starling RC, editors. Mechanical Support for Heart Failure. Cham: Springer International Publishing. p. 707–28.

[12] Pauls JP Stevens MC Bartnikowski N Fraser JF Gregory SD Tansley G. Evaluation of physiological control systems for rotary left ventricular assist devices: an *in-vitro* study. Ann Biomed Eng. (2016) 44:1–11. 10.1007/s10439-016-1552-3 26833037

[13] Petrou A Lee J Dual S Ochsner G Meboldt M Schmid Daners M. Standardized comparison of selected physiological controllers for rotary blood pumps: *in vitro* study. Artif Organs. (2018) 42:E29–42. 10.1111/aor.12999 29094351

[14] Schima H Vollkron M Jantsch U Crevenna R Roethy W Benkowski R. First clinical experience with an automatic control system for rotary blood pumps during ergometry and right-heart catheterization. J Hear Lung Transplant. (2006) 25:167–73. 10.1016/j.healun.2005.09.008 16446216

[15] Pauls JP Roberts LA Burgess T Fraser JF Gregory SD Tansley G. Time course response of the heart and circulatory system to active postural changes. J Biomech Eng. (2018) 140:034501. 10.1115/1.4038429 29131882

[16] Looga R. The Valsalva manoeuvre - cardiovascular effects and performance technique: a critical review. Respir Physiol Neurobiol. (2005) 147:39–49. 10.1016/j.resp.2005.01.003 15848122

[17] Granegger M Masetti M Laohasurayodhin R Schloeglhofer T Zimpfer D Schima H. Continuous monitoring of aortic valve opening in rotary blood pump patients. IEEE Trans Biomed Eng. (2016) 63:1201–7. 10.1109/TBME.2015.2489188 26461795

[18] Maw M Gross C Schlöglhofer T Dimitrov K Zimpfer D Moscato F. Development of suction detection algorithms for a left ventricular assist device from patient data. Biomed Signal Process Control. (2021) 69:102910. 10.1016/j.bspc.2021.10291023192602

[19] Moscato F Granegger M Edelmayer M Zimpfer D Schima H. Continuous monitoring of cardiac rhythms in left ventricular assist device patients. Artif Organs. (2014) 38:191–8. 10.1111/aor.12141 23902542

[20] Pan J Tompkins WJ. Pan tomkins 1985 - QRS detection.pdf. IEEE Trans Biomed Eng. (1985) 32:230–6. 10.1109/TBME.1985.325532 3997178

[21] Pardey J Jouravleva S. The next-generation holter revolution: From analyse-edit-print to analyse-print. Comput Cardiol. (2004) 31:373–6. 10.1109/CIC.2004.1442950

[22] Schlöglhofer T Aigner P Migas M Beitzke D Dimitrov K Wittmann F. Inflow cannula position as risk factor for stroke in patients with HeartMate 3 left ventricular assist devices. Artif Organs. (2022). 10.1111/aor.14165. [Epub ahead of print]. 34978722 PMC9305857

[23] Vollkron M Voitl P Ta J Wieselthaler G Schima H. Suction events during left ventricular support and ventricular arrhythmias. J Hear Lung Transplant. (2007) 26:819–25. 10.1016/j.healun.2007.05.011 17692786

[24] Gopinathannair R Cornwell WK Dukes JW Ellis CR Hickey KT Joglar JA. Device therapy and arrhythmia management in left ventricular assist device recipients: a scientific statement from the American Heart Association. Circulation. (2019) 139:E967–89. 10.1161/CIR.0000000000000673 30943783

[25] Hayward CS Salamonsen R Keogh AM Woodard J Ayre P Prichard R. Effect of alteration in pump speed on pump output and left ventricular filling with continuous-flow left ventricular assist device. ASAIO J. (2011) 57:495–500. 10.1097/MAT.0b013e318233b112 21989420

[26] Sorensen EN Kon ZN Feller ED Pham SM Griffith BP. Quantitative assessment of inflow malposition in two continuous-flow left ventricular assist devices. Ann Thorac Surg. (2018) 105:1377–83. 10.1016/j.athoracsur.2017.12.004 29305851

[27] Vincent F Rauch A Loobuyck V Robin E Nix C Vincentelli A. Arterial pulsatility and circulating von willebrand factor in patients on mechanical circulatory support. J Am Coll Cardiol. (2018) 71:2106–18. 10.1016/j.jacc.2018.02.075 29747831

[28] Lai J V Muthiah K Robson D Prichard R Walker R Pin Lim C. Impact of pump speed on hemodynamics with exercise in continuous flow ventricular assist device patients. ASAIO J. (2020) 66:132–8. 10.1097/MAT.0000000000000975 30913099

[29] Jakovljevic DG George RS Nunan D Donovan G Bougard RS Yacoub MH. The impact of acute reduction of continuous-flow left ventricular assist device support on cardiac and exercise performance. Heart. (2010) 96:1390–5. 10.1136/hrt.2010.193698 20643664

[30] Jain P Adji A Emmanuel S Robson D Muthiah K Macdonald PS. Phenotyping of stable left ventricular assist device patients using noninvasive pump flow responses to acute loading transients. J Card Fail. (2021) 27:642–50. 10.1016/j.cardfail.2021.01.009 33497807

[31] Vollkron M Schima H Huber L Benkowski R Morello G Wieselthaler G. Development of a reliable automatic speed control system for rotary blood pumps. J Hear Lung Transplant. (2005) 24:1878–85. 10.1016/j.healun.2005.02.004 16297795

