# Supplementary material for: A Sensorless Modular Multiobjective Control Algorithm for Left Ventricular Assist Devices: A Clinical Pilot Study
Source: Front Cardiovasc Med. 2022 Apr 25;9:888269. doi: 10.3389/fcvm.2022.888269 (PMC9081924; doi:10.3389/fcvm.2022.888269)
Supplement: Supplementary file 1 [file Data_Sheet_1.pdf]

Supplementary Material

S1: Detailed description of the control system

The PhC control system consists of modules which are supplied with pump data and clinician setpoints. (Figure S-1) In the following section the modules are described in more detail.

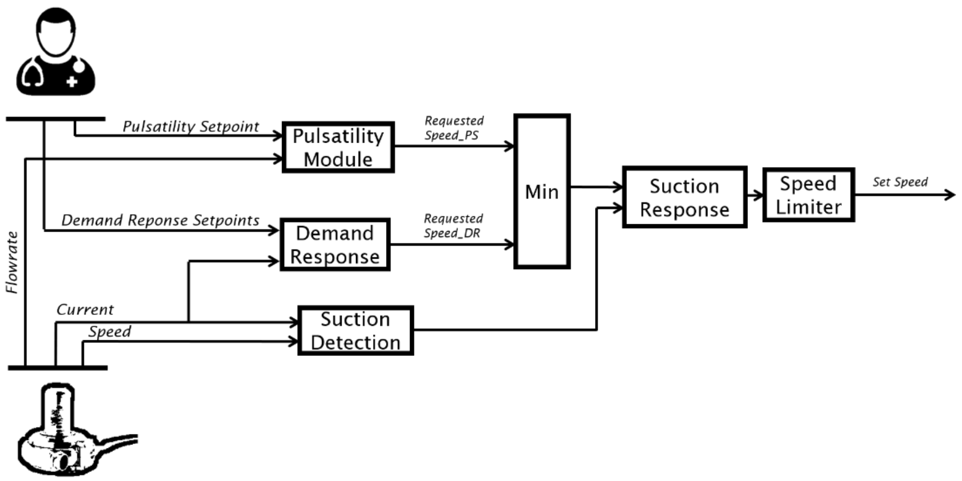

Figure S1-1: Control modules and their interaction. Setpoints are supplied to the demand response and pulsatility module. Pump signals are distributed to the modules. The minimum of demand response and pulsatility module suggested speeds is passed through the suction response module, which decreases speed upon encountering suction and the speed limiter module which limits absolute and relative set speeds.

Demand response module

This module calculates an instantaneous HR estimate from pump current. To gain a more robust estimate, a median filter with a window length of 12 beats is used.

Desired pump speed is set as a linear function of HR defined by 2 points, termed Rest and Exercise desired speed. Nonphysiologic increases in heart rate or highly variable RR intervals, indicative of arrhythmia, disable the link between heartrate and desired speed and set the desired speed to the rest speed. Insufficient pulsatility to determine HR also set the desired pump speed to the resting speed.

Pulsatilty module

Flowrate pulsatility is calculated over a 2 seconds window from the estimated pump flowrate. As pulsatility only becomes a suitable surrogate of preload at low pulsatilities, target flowrate setpoint is set approximately 1.5 L/min below usual pulsatility during sitting. An Integral

controller with a gain of 60 $rpm/\frac{L}{min}/s$ is used to maintain pulsatility level, should pulsatility drop beyond this point.

Suction detection and reaction module.

Suction is detected from pump signals via a novel binary suction detection algorithm, based on an adaptive thresholding approach, which achieved high per-beat sensitivity and specificity in a previously presented clinical dataset. (per beat, test-set Specificity: 97.7% Sensitivity 88.2%) [18] (see S2 in supplemental material)

Suction reaction rules were set such that speed is reduced by a fixed 100 rpm if one of a set of conditions apply such as suction present in 3/5 seconds, 4/6 or 8/20. If suction is not encountered in more than a single beat in the 12 seconds following a decrease or increase, speed is increased again by 100 rpm. Speed may be reduced to a user defined minimum.

Supervision module

The lower pump speed always has priority, such that speed may not be increased above the DR-Module output even with sufficient pulsatility. Suction Reaction module is enabled once sufficient suction is detected and is disabled again when either PS or DR speeds are lower than Suction Response suggested speed.

Pump set-speeds sent to the controller must adhere to absolute and relative safety limits, which were determined on current clinical practice. No set-speeds lower than 2000 rpm and higher than 3400 rpm may be sent to the controller and no relative changes of more than 200 rpm/10 seconds. The Lavare© Cycle may bypass those restrictions.

The Speed-Current Ratio (SCR) algorithm

The algorithm uses two signals, pump speed and current, to generate the speed-current ratio (SCR) waveform.

Speed-Current Ratio (SCR) = Speed/Current

Where speed is the estimated pump speed (units = RPM) and current is the pump input current (units = mA).

Adaptive threshold feature

The adaptive threshold feature adjusts the SCR threshold based on a baseline and pulsatility calculated from recent values of the SCR, contained in a 5 second rolling buffer. Only non-suction beats are admitted to the buffer.

Pulsatility(n) = 80th percentile SCR– 20th percentile SCR

The baseline(*n*) for sample *n* is the 60th percentile SCR value in the buffer.

$$Threshold(n) = Baseline(n) + Pulsatility(n)*1.3$$

Two consecutive samples above the threshold are considered suction and are excluded from admission to the rolling buffer.

S-2: Demographic and individual test tables

Supplemental Table S2-1:

Patient Demographics. IC Angle = Inflow Cannula Angle, Medication: AB= Alpha Adrenic Blocking Agents, ACE-I = Angiotension Conversion Enzyme Inhibitor, AM = Amiodaron, BB= Beta Adrenic Blocking agents, CCB = Calcium Channel Blockers, L= Loop Diuretics, P=Phencromoumon. Comorbitities: AI = Aortic Insufficiency, ARDS = Acute Respiratory Distress Syndrome, COPD = chronic obstructive pulmonary disease, MC = Myocarditis, MD = Muscular Dystrophy

Pat	Sex	Age	Ischemic Etiology	Weight (kg)	Height (m)	CRT	Intermacs	LVAD Indication	Minimal Invasive?	IC Angle (°)	Medication	Comorbidity
**1**	m	68	Y	87	1,79	y	3	BTC	n	20	ACE-I, BB, L, P	COPD, Renal Insufficiency
**2**	m	40	n	128	1,93	n	2	BTT	n	-16	ACE-I, AM, L, P	n
**3**	m	51	y	77	1,72	n	4	BTC	n	-30	ACE-I, AM, BB, L, P	n
**4**	m	56	y	124	1,78	n	4	BTC	y	32	AB, BB, L, P	Diabetes, COPD
**5**	m	56	y	96	1,84	n	1	BTC	n	9	ACE-I, BB, L, P	AI, Renal Insufficiency
**6**	m	51	n	109	1,76	y	3	BTC	y	-5	AM, AB, BB, CCB, L, P	ARDS, MC, Epilepsy, MD

Supplemental Table S2-2:

Overview of the tests: Identifier contains patient tag (Pat), post operative day (POD) and test setting, which was either intermediate care unit (IC) or ambulatory ward (AM). Premeasurement evaluations consist of constant speed setspeed (CS ω), aortic valve opening(AoV) can either be always (1), intermittent (0.5) or not opening (0), end diastolic diameter (EDD), heart rate (HR), mean arterial pressure (MAP), pump flowrate (Q) and flowrate pulsatility (Qp2p). Controller setpoints include target pulsatility (Set_Qp2p), rest and exercise setpoints for heart rate (HR_Rest, HR_Ex) and speed (ω_Rest, ω_Ex), which are set according to the setpoint strategy: "Protocol" means set according to the protocol outlined in method section, "Pulsatility" means increased target pulsatility settings and high DR denotes elevated HR setpoints. The first column in the measurement section denotes which mode the patient completed first. Qp2pRange shows the pulsatility range throughout all measurements. Similarly, HR_Range gives the range of HR. OR Type denotes the type of orthostatic transition that the patients were able to complete H: patients were able to stand, M: Patients were able to sit. VA and ERGO denote the availability of comparable data for valsalva maneuver and ergometry respectively. Finally, ECG lists whether Holter ECG was available for a given test.

Identifier				Premeasurement							Controller Setpoints						Measurements						
**Trial**	Pat	POD	Setting	CS ω	AoV	EDD	HR	MAP	Q	Qp2p	Set Qp2p	HR Rest	HR Ex	ω Rest	ω Ex	Set.Strat.	First	Qp2p Range	HR Range	OR Typ	VA	ERGO max	ECG
**1**	1	18	IC	2700	0		85	70	4,3	1	2	80	100	2500	3000	Pulsatility	CS	0.5-3.0	81-97	-	-	Y	-
**2**	2	932	AM	2700	0,5	60	80	86	4,9	5,5	3	80	100	2500	3000	Protocol	PhC	2.5-6.0	65-107	H	y	45	-
**3**	3	12	IC	2700	1	62	103	73	5	3,8	2,5	90	110	2500	2900	Protocol	CS	3.0-4.5	103-107	H	y	-	-
**4**	3	18	IC	2700	1	61,3	100	64	5,2	3,5	2,5	90	110	2500	2900	Protocol	PhC	2.0-4.5	100-107	M	-	Y	-
**5**	2	988	AM	2700	1	67	68	80	5,4	5	3	65	110	2500	3000	Protocol	PhC	2.5-6.5	62-120	M	y	45	-
**6**	1	115	AM	2700	0	55,6	55	65	4,9	0,5	1,2	60	100	2500	2900	Pulsatility	PhC	0.0-4.0	55-93	H	y	25	y
**7**	3	76	AM	2600	1	35	59	86	4,6	4,5	2	65	100	2500	2900	Protocol	PhC	3.5-5.5	62-88	H	y	60	-
**8**	5	266	AM	2700	0	85	61	85	5,4	2,5	2,5	60	90	2460	2960	Pulsatility	CS	2.0 -3.0	62-83	H	y	35	y
**9**	4	268	AM	2500	1	49	71	75	4,5	4,7	3	60	90	2400	2800	Protocol	CS	4.0-6.5	60-90	H	y	50	y

**10**	6	693	AM	2860	0	54	100	100	5,4	2,5	2	100	120	2700	3000	High DR	PhC	1.0-3.5	100-107	M	y	-	y
**11**	5	294	AM	2700	1	64	60	84	4,9	2,6	1,5	60	75	2500	3100	High DR	CS	2.0-3.0	58-81	H	y	60	y
**12**	2	1073	AM	2700	1	65	75	75	5,5	4,5	3	65	110	2500	3200	High DR	PhC	4.0-6.5	77-120	M	y	40	y
**13**	1	178	AM	2600	0	62	80	84	4,5	4,5	2	60	85	2860	3060	High DR	PhC	0.0-4.5	55-100	H	y	40	y

S3: Standardized protocol results: Box Plots with single traces denoting individual tests. These figures are extensions of the main figures 3-6.

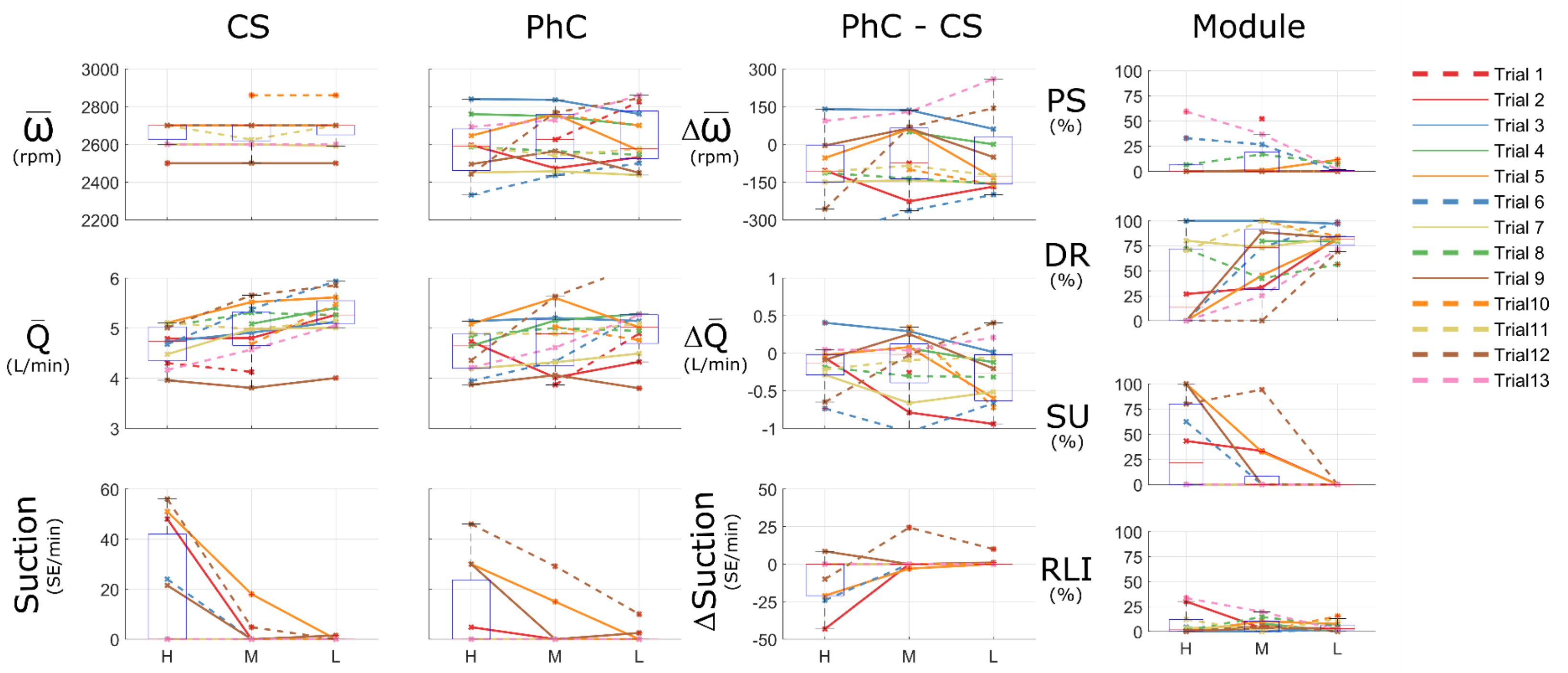

Figure S3-1: Steady state posture differences between H (standing), M (sitting), L (supine). Left panels: Speed(ω), flowrate(Q), and suction in constant speed (CS) and physiologic control (PhC) and their differences. Module panel: control module activation for pulsatility (PS), Demand response (DR), suction response (SU), and rate limited increase (RLI) modules. Colored lines indicate the separate tests. Dashed lines indicate trials with pulsatility setpoint focus, solid lines indicate trials with setpoints per-protocol.

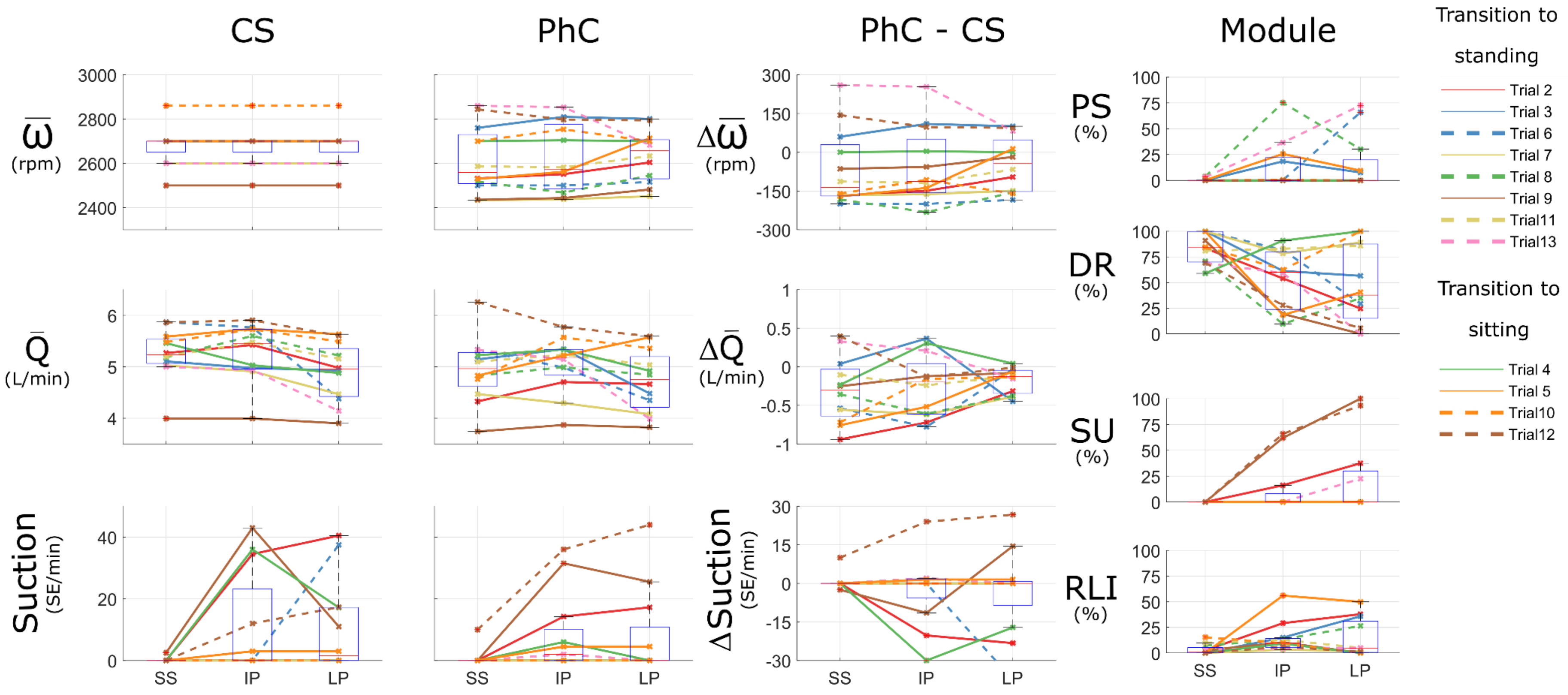

Figure S3-2: Orthostatic transitions from the supine(L) position to either sitting (M) or standing(H). Left panels: speed(ω), flowrate(Q), and suction in constant speed (CS) and physiologic control (PhC) and their differences are presented in steady state (SS) initial transition phase (IP) and late phase (LP). Module panel: control module activation: activation for pulsatility (PS), demand response (DR), suction response

(SU), and rate limited increase (RLI) modules. Colored lines indicate the separate tests. Dashed lines indicate trials with pulsatility setpoint focus, solid lines indicate trials with setpoints per-protocol.

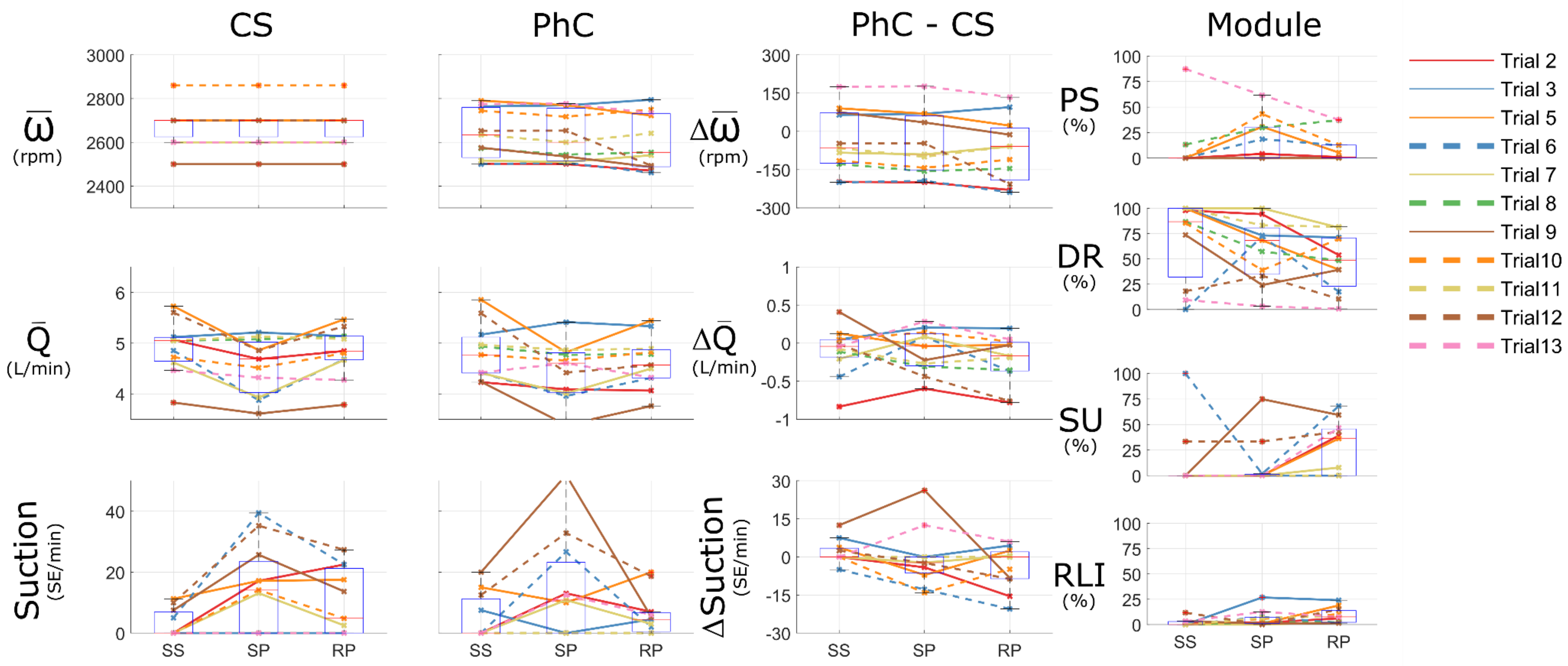

Figure S3-3: Valsalva maneuver(VA) is subdivided into 3 phases: steady state(SS), strain phase(SP) and recovery phase (RP). Left panels: speed(ω), flowrate(Q), and suction in constant speed (CS) and physiologic control (PhC) and their differences are presented. Module panel: controlodule activation: activation for pulsatility (PS), demand Response (DR),suction response (SU), and rate limited increase (RLI) modules. Colored lines indicate the separate tests. Dashed lines indicate trials with pulsatility setpoint focus, solid lines indicate trials with setpoints per-protocol.

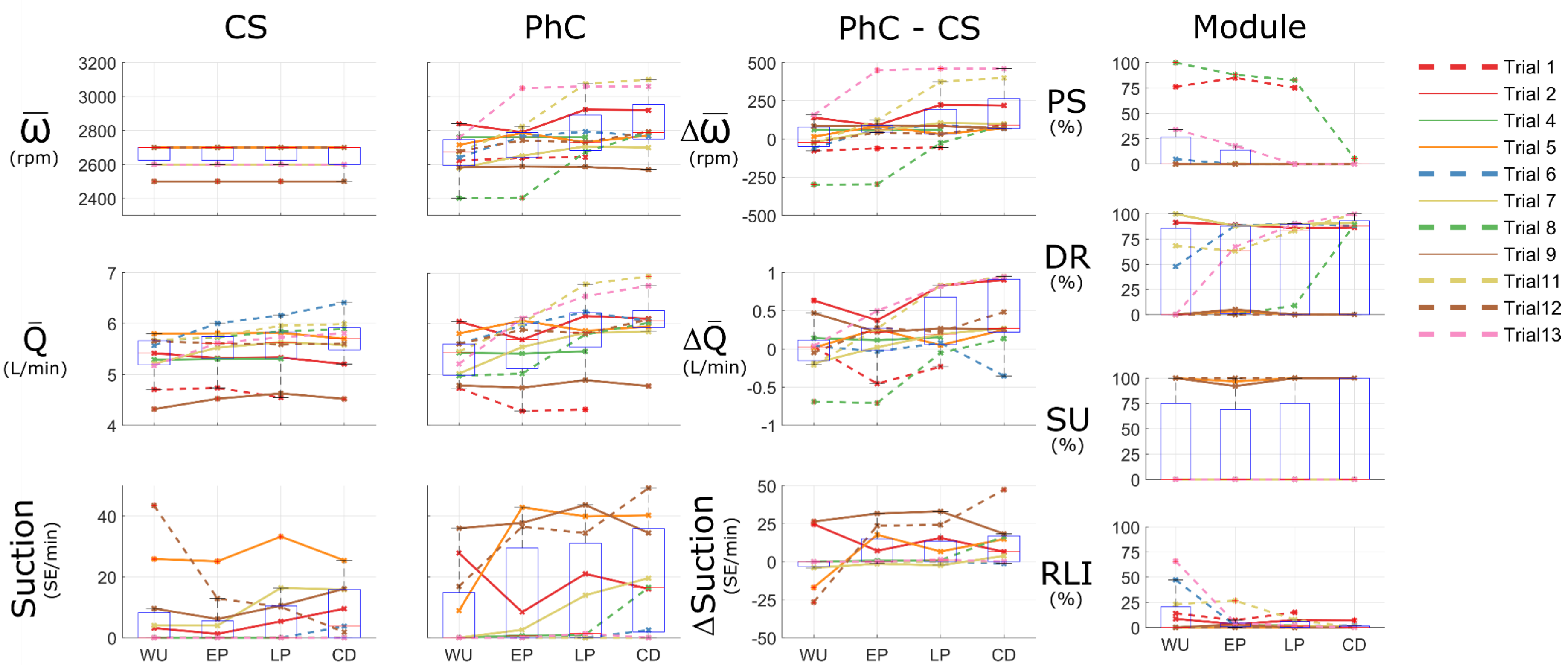

Figure S3-4: Ergometry is subdivided into 4 phases: warm up (WU), early phase(EP), late phase (LP) and cool down (CD). Left Panel: speed(w), flowrate(Q), and suction in constant speed (CS) and physiologic control (PhC) and their differences are presented. Module panel: control module activation: activation for pulsatility (PS), demand response (DR), suction response (SU), and rate limited increase (RLI) modules. Colored lines indicate the separate tests. Dashed lines indicate trials with pulsatility setpoint focus, solid lines indicate trials with setpoints per-protocol.

S4-S6: Selected snapshots of the maneuvers

Upper panel shows a median filtered heartrate in bpm. Second panel shows pump speed. Third panel displays instantaneous estimated flowrate as well as low pass filtered flowrate. Lower panel shows activated modes, Lavare activity, Suction classification result of the reference classifier and the novel SCR as well as aortic valve opening.

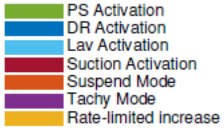

Figure S4-6-1: Legend for bottom panel “Modes”

S4: Single Snapshots and Waveforms of Orthostatic Maneuvers

Test 2 (Figure S4-1) shows an example of averted suction by PhC due to lower speed at maneuver onset. Activation of the pulsatility module prevents suction by reducing speed at low pulsatility in test 6 in figure S4-2). Higher Suction Burden in PhC was caused either by increased speed at steady state (test 12, Figure S4-4) or repeated attempts at speed increase at low speeds (<2500 rpm), which retriggered suction events (test 9, Figure S4-3).

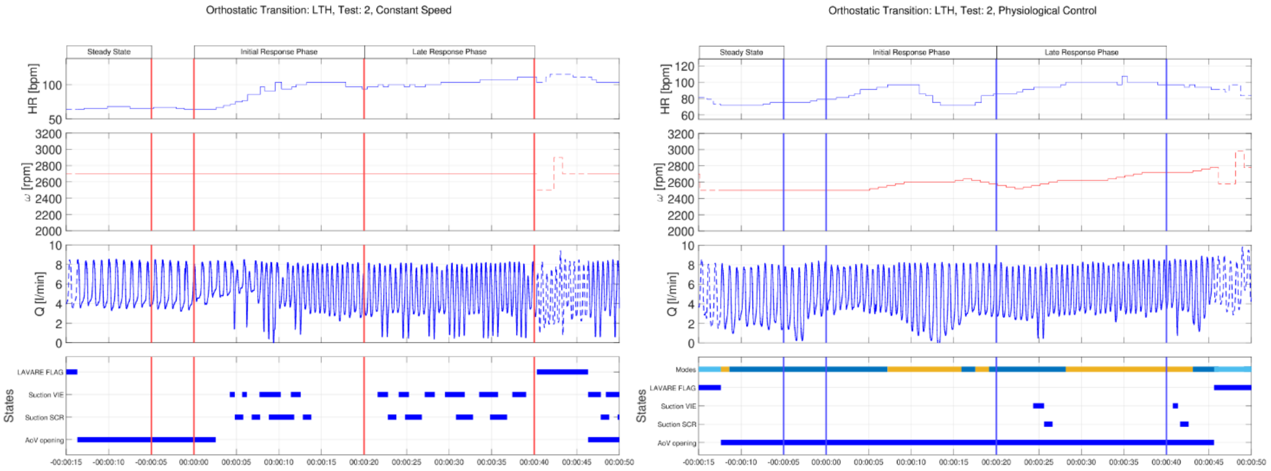

Figure S4-1: Test 2 lower baseline speed averts suction during maneuver.

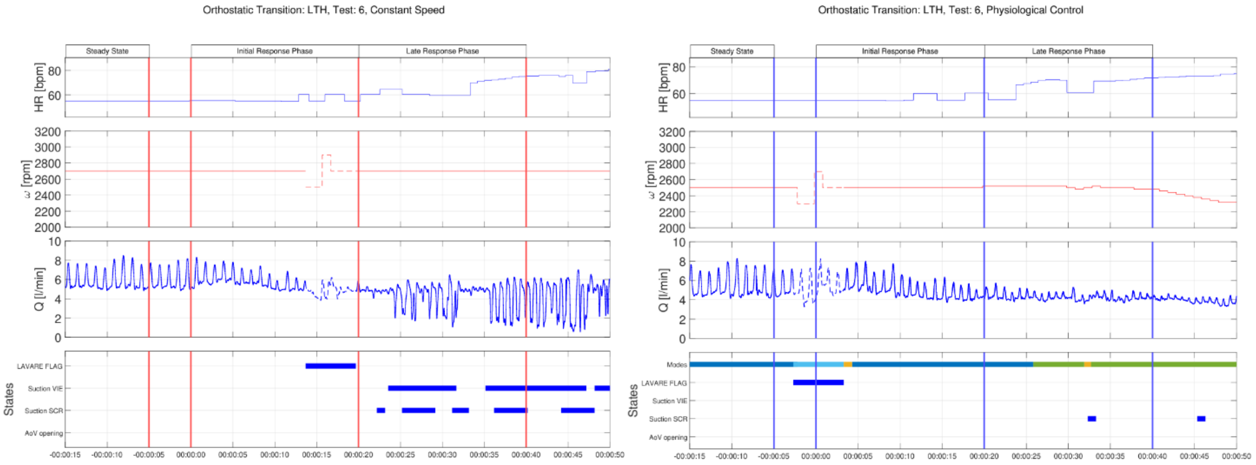

Figure S4-2: in test 6, lower speed at onset and speed reduction due to insufficient pulsatility reduced suction burden.

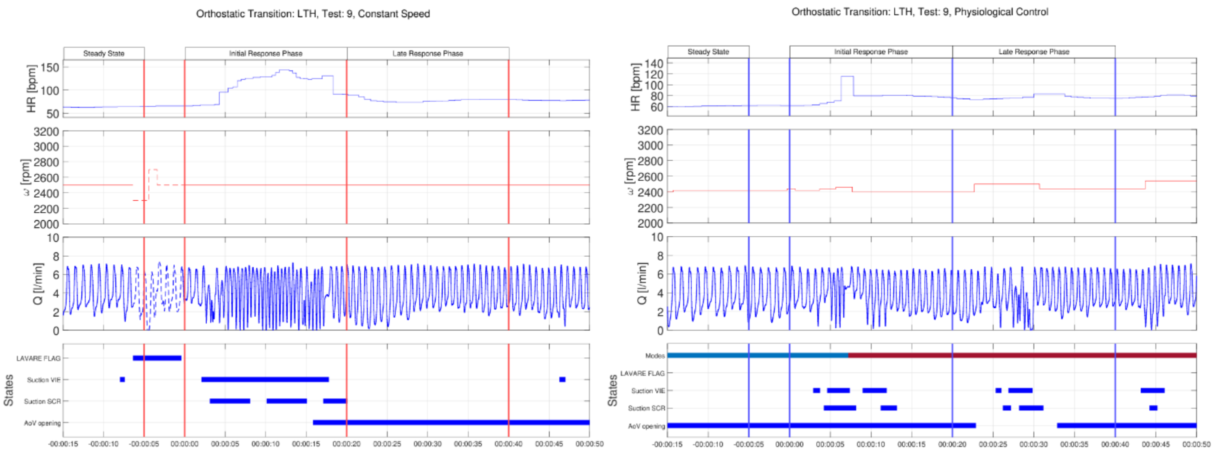

Figure S4-3: in test 9, repeated attempts at speed increase at low speeds retriggers suction. Patient has a low CS set point.

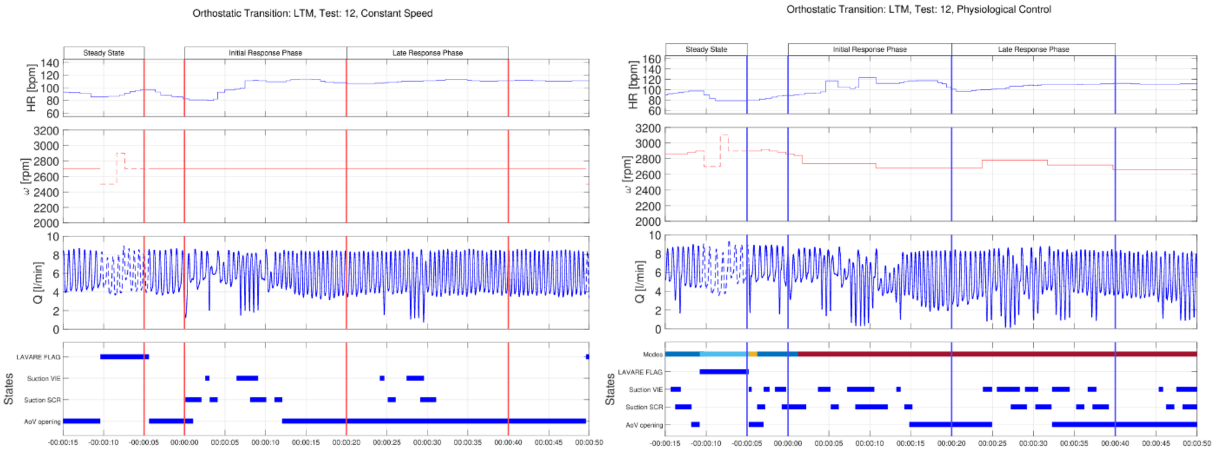

Figure S4-4: In test 12, increased speed at steady state leads to higher suction burden du to insufficient speed of reaction.

S5: Single Snapshots and Waveforms of Valsalva Maneuver

In some Valsalva maneuvers, there was little physiologic response, an example is given in Figure S5-1. In most tests suction burden is reduced due to the reduced speed at onset or module activation, an example is given in figure S5-2. Increased speed at baseline in test 9 and 13 with insufficiently fast response is given in figures S5-3 and S5-4 respectively.

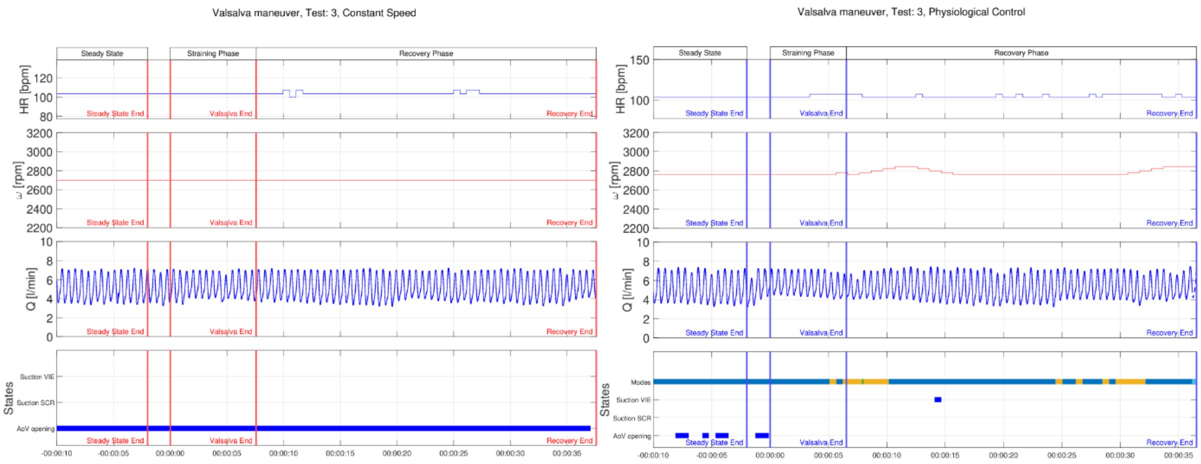

Figure S5-1: no response to Valsalva maneuver example in test 3.

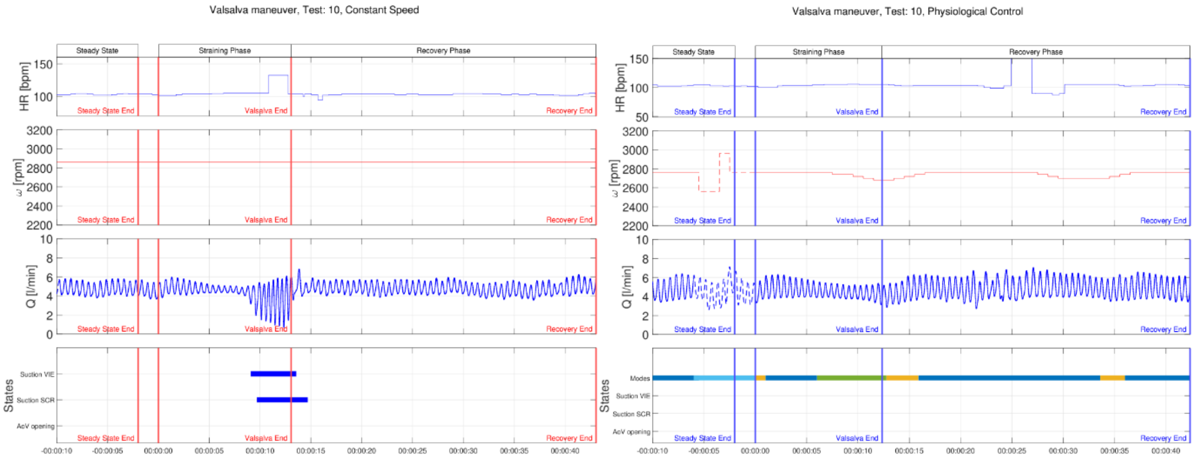

Figure S5-2: Suction burden is reduced due to reduced speed at onset and PS activation in test 10

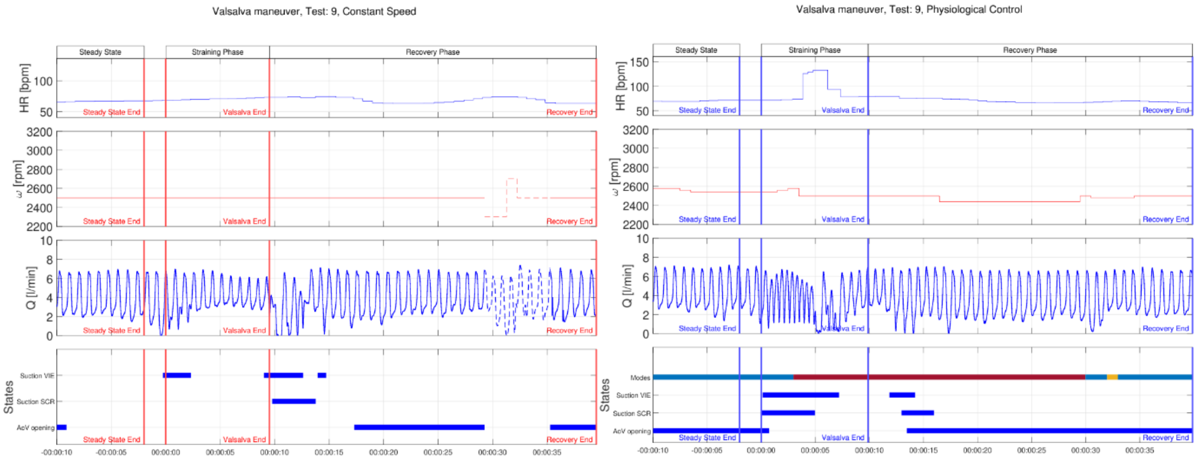

Figure S5-3: Suction at low speeds with increased speed compared to CS in test 9.

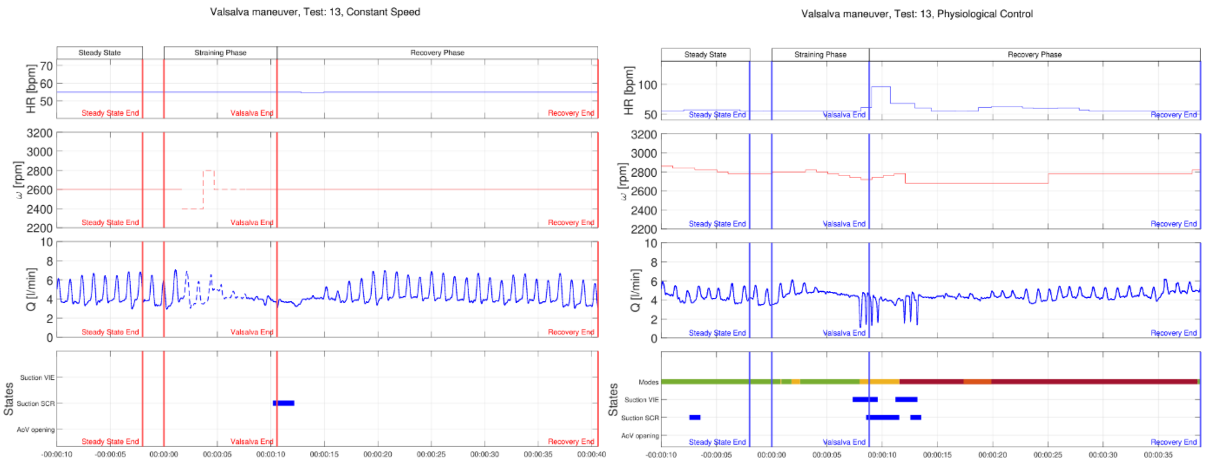

Figure S5-4: Suction is increased due to increased speed at baseline and rate limited response in test 13.

S6: Single Snapshots and waveforms of ergometry

Persistent suction upon speed increase (test 9, fig. S6-4) or a complete chronotropic incompetence and lack of pulsatility response shortly after implantation (test 1 and 4, Fig S6-1 and & S6-2 respectively) restricted speed increase. In one test, speed was only increased in the late phase, as pulsatility remained low until then (test 8, fig. S6-3).

There was no marked decrease in flowrate pulsatility before suction in these patients (minimum pulsatility > 3.5). The reasons for the suction increase were suction classifier discrepancy (test 2), or repeated unsuccessful attempts at speed increase past CS setpoint in test 9 and 12, (figs. S6-6 and S6-7)) which were by pat 4 and 2 with cannula inflow angles of 32° and -15° respectively. In the 2 tests where speed was increased the most (>300 rpm) (tests 11 and 13, fig S6-5) suction burden remained below 3 SE/min in both modes. In these patients (Pat. 1 and 5) cannula angles were more favorable at 20° and 9° respectively.

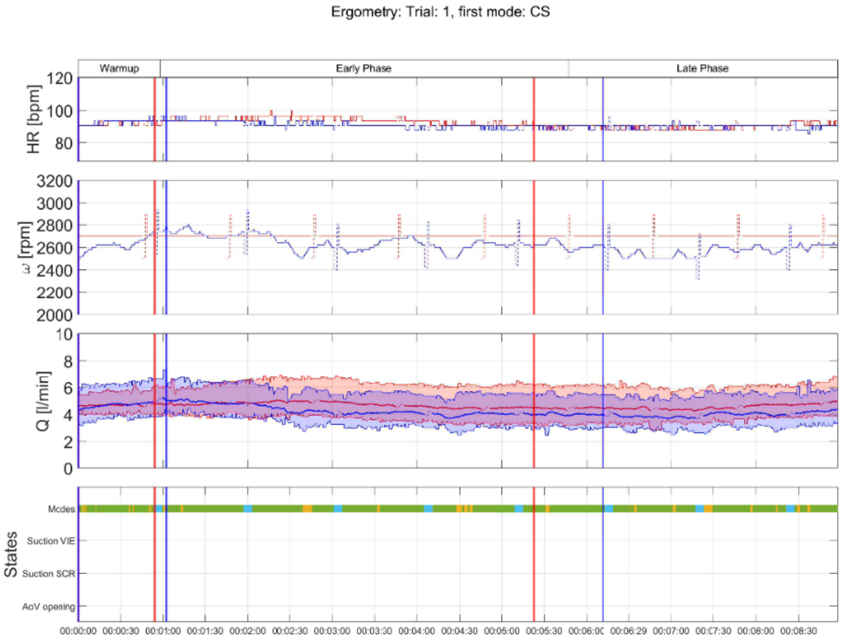

Figure **S6-1:** In test 1, no pulsatility and very little HR response was observed.

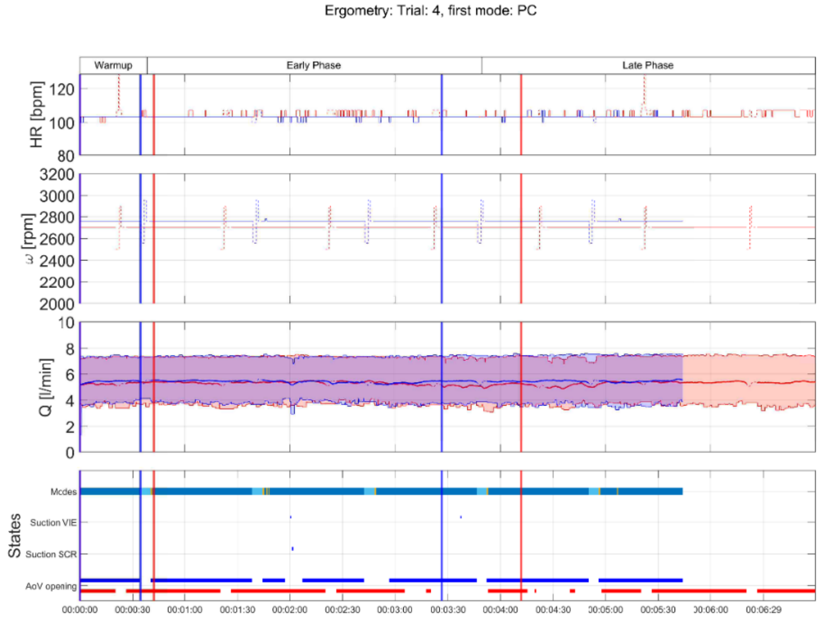

Figure **S6-2**: In test 4 there was no HR response and no change in pulsatility.

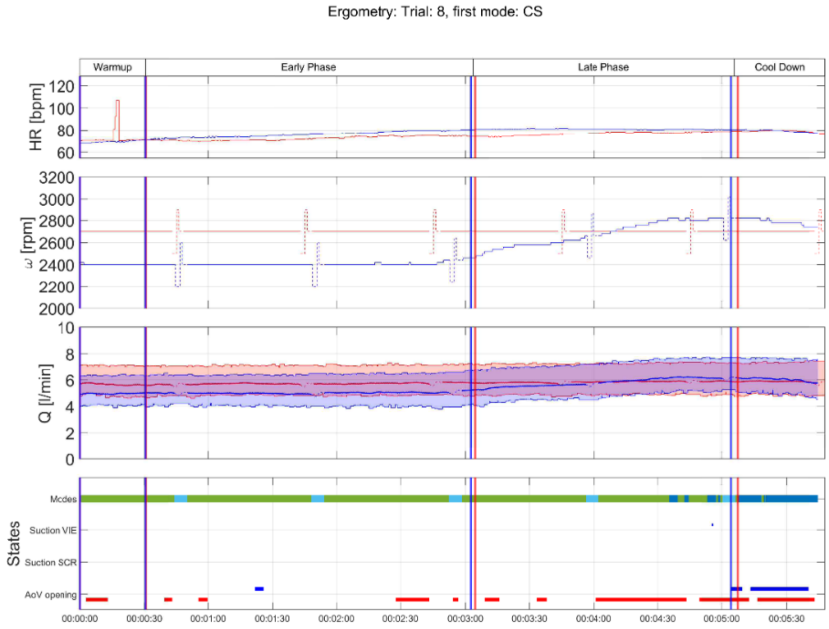

Figure **S6-3**: In test 8, pulsatility only increased in the later stages of exercise, limiting speed increase before then.

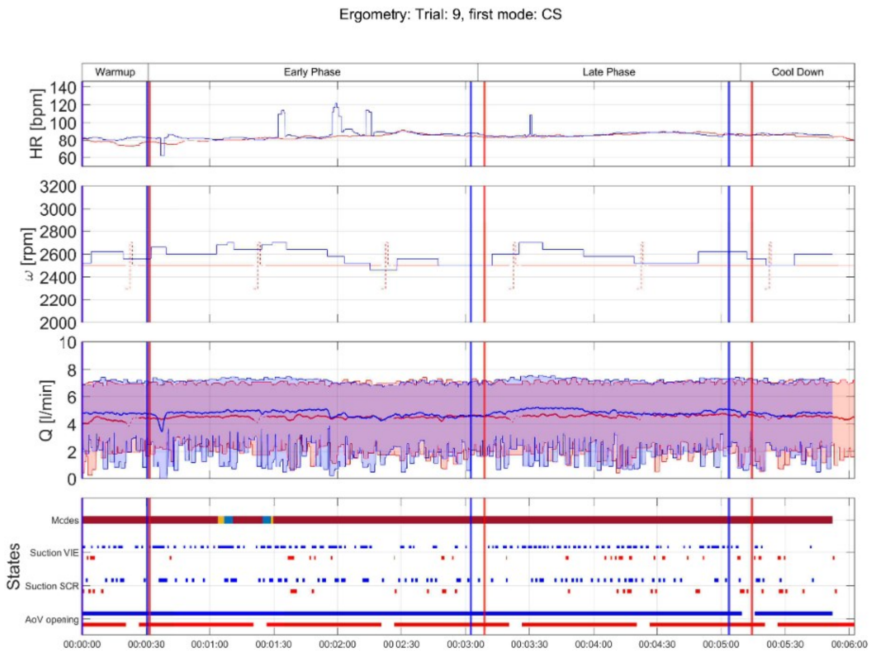

Figure **S6-4**: Repeated attempts at speed increase above CS set speed result in higher suction rates in test 9.

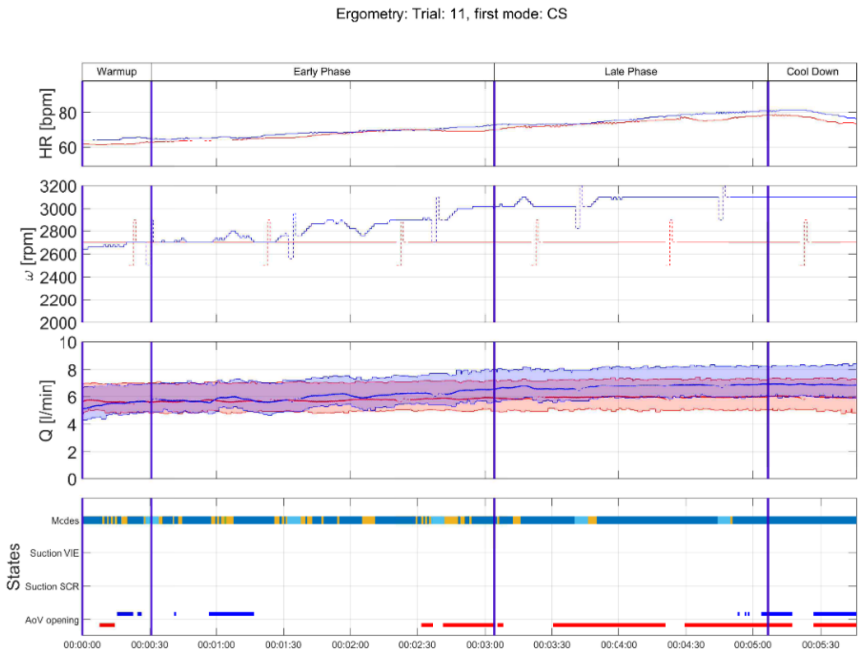

Figure **S6-5**: In Trial 11 speed is increased by 400 rpm over CS set speed during late phase of submaximal exercise.

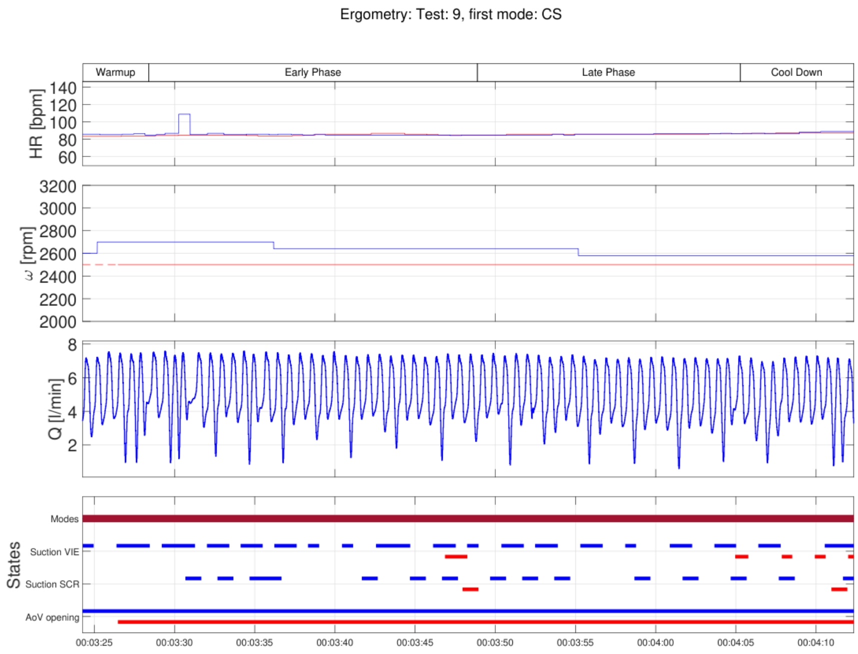

Figure **S6-6**: intermittent suction persisted even at later stages of submaximal exercise for test 9.

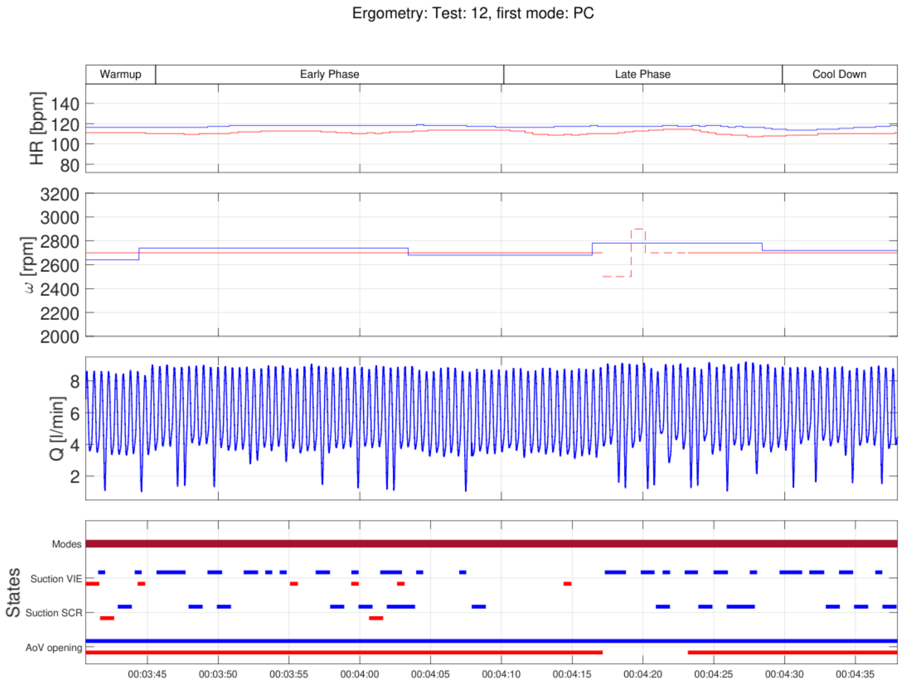

Figure **S6-7**: repeated attempts at speed increase in test 12 resulted in retriggered suction events during later stages of submaximal exercise.
